# The Phytogeographic History of Common Walnut in China

**DOI:** 10.3389/fpls.2018.01399

**Published:** 2018-09-21

**Authors:** Xiaojia Feng, Huijuan Zhou, Saman Zulfiqar, Xiang Luo, Yiheng Hu, Li Feng, Maria E. Malvolti, Keith Woeste, Peng Zhao

**Affiliations:** ^1^Key Laboratory of Resource Biology and Biotechnology in Western China, Ministry of Education, College of Life Sciences, Northwest University, Xi’an, China; ^2^Zhengzhou Fruit Research Institute, Chinese Academy of Agricultural Sciences, Zhengzhou, China; ^3^Institute of Agro-environmental and Forest Biology, Consiglio Nazionale delle Ricerche, Terni, Italy; ^4^USDA Forest Service Hardwood Tree Improvement and Regeneration Center (HTIRC), Department of Forestry and Natural Resources, Purdue University, West Lafayette, IN, United States

**Keywords:** microsatellites, *Juglans regia*, Persian walnut, genetic structure, chloroplast, ecological niche model, migration, refugia

## Abstract

Common walnut (*Juglans regia* L.) is an economically important hardwood tree species cultivated worldwide for its high quality wood and edible nuts. It is generally accepted that after the last glaciation *J*. *regia* survived and grew in almost completely isolated stands in Asia, and that ancient humans dispersed walnuts across Asia and into new habitats via trade and cultural expansion. The history of common walnut in China is a matter of debate, however. We estimated the genetic diversity and spatial genetic structure of 31 walnut populations sampled across its Chinese range using 22 microsatellite markers (13 neutral and 9 non-neutral). Using historical data and population genetic analysis, including approximate Bayesian analysis (ABC), we reconstructed the demographic history of *J*. *regia* in China. The genetic data indicated the likely presence of *J*. *regia* in glacial refugia in the Xinjiang province (Northwest China), Northeastern China (Beijing, Shandong, and Changbai Mountains), Central China (Qinling and Baishan Mountains and Xi’an), and Southwestern China (Tibet, Yunnan, Guizhou, and Sichuan provinces). Based on DIY-ABC analysis, we identified three ancient lineages of *J. regia* in China. Two lineages (subpopulation A and subpopulation B+C) diverged about 2.79 Mya, while Southwestern China, and Qinling and Baishan Mountains lineages diverged during the Quaternary glaciations (about 1.13 Mya). Remnants of these once-distinct genetic clusters of *J. regia* may warrant ecological management if they are to be retained as *in situ* resources. A population size expansion in Northeastern China was detected in the last five centuries. The present distribution of walnut in China resulted from the combined effects of expansion/contraction from multiple refugia after the Last Glacial Maximum and later human exploitation.

## Introduction

Persian or common walnut (*Juglans regia* L.) is an economically important hardwood tree species cultivated worldwide for its high quality wood and edible nuts (Manning, 1978; Chen et al., 2014; Han et al., 2016; Pollegioni et al., 2017). *J. regia* is diploid (Woodworth, 1930), monoecious and heterodichogamous. It is wind pollinated and highly heterozygous (Manning, 1978; Dangl et al., 2005). The native range of common walnut is uncertain, but (apparently) wild populations grow in isolated favorable habitats across a wide geographical range from China to the Iberian Peninsula (Manning, 1978; Draine and Hiden, 1998; Beer et al., 2008; Martínez-García et al., 2016; Pollegioni et al., 2017). Because of continued uncertainty concerning the number and locations of refugia for *J. regia*, current populations may or may not have been derived from refugia in Southern Europe, the Balkans, the Carpathian mountains, Anatolia, the Zagros mountains, and even China (Martínez-García et al., 2016; Aradhya et al., 2017).

*Juglans regia* is considered one of the earliest tree foods used by humans; historical references to its use date back to Persia (7,000 BCE) (Renault-Miskovsky et al., 1984; Beer et al., 2008). The evolutionary history of common walnut (*J. regia*) in Asia has emerged as a complex interaction of biogeography, climate change, and human forces (Pollegioni et al., 2015, 2017); the same forces have likely shaped the evolution of common walnut in China (Hu et al., 2014; Han et al., 2016). Humans traded walnuts along the Silk Road and Persian Royal Road, dispersing common walnut genes across imposing natural barriers and across long geographic distances (Vahdati, 2013; Pollegioni et al., 2017). For example, the northern route of the northern Silk Road originated from the historical capital of Chang’an (now Xi’an, Shaanxi province), ran through Gansu province via Lanzhou and Dunhuang along the Hexi Corridor (Pollegioni et al., 2015, 2017). This road led westward along the northern foothills of the Eastern Tien Shan Mountains. It connected Eastern China (Shandong province) and Western China (Eastern Tien Shan mountains; Urumqi; Xinjiang province), facilitating cultural and agrarian exchange (Christian, 2000; Aradhya et al., 2017; Pollegioni et al., 2017).

Phylogeography seeks to understand the evolutionary history and distribution of organisms (Qian and Ricklefs, 2000; Sechrest et al., 2002; Beheregaray, 2008; Avise, 2009). Phylogeography has been a focus of evolutionary biology. Of particular importance to Chinese phylogeography (Qiu et al., 2011; Liu et al., 2012) is the Qinghai-Tibetan Plateau (QTP) (Qiu et al., 2011; Wen et al., 2014; Favre et al., 2015; Hughes and Atchison, 2015), Southwestern China (Fan et al., 2013; Du et al., 2017), and Northern China (Chen et al., 2008; Zeng et al., 2011). The phylogeography of *Juglans* in China and adjacent areas has been the basis of several recent studies (Bai et al., 2010, 2014, 2016; Wang et al., 2016). The spatial and geographic distribution of genetic variation of *Juglans* species has been investigated at the intraspecific level and among closely related species in South China (Bai et al., 2014; Wang et al., 2015; Han et al., 2016). These and other studies shed light on the complex interactions of geography, ecology, and changing climate in shaping biogeography (Fan et al., 2013; Du et al., 2017). For most plant species analyzed, the best explanation for their phylogeography entails multiple glacial refugia (Qiu et al., 2011; Zeng et al., 2011; Liu et al., 2012; Shi et al., 2014), but there is no consensus yet concerning the locations and species composition of glacial refugia in China, reducing the impact of conclusions (Chen et al., 2012).

Common walnut was part of the ancient Chinese flora too; ^14^C-dated leaf fossils and carbonized nuts found in Shandong and Hebei provinces were ca. 7,335 ± 100 years old. Other similar samples have been recovered from Henan province (7,200 ± 80 years old), and Shaanxi (∼6,000 years old) (Xi, 1989). The timing, nature, and extent of the use of *J. regia* by humans in China have been a subject of debate among foresters, botanists, biogeographers, and other scientists (Xi, 1989). Among several theories, the most popular is that Zhang Qian (a Chinese official and diplomat who served as an imperial envoy to the world outside of China in the 2nd century BC, during the Han Dynasty) introduced walnut into China 2,100 years ago (Xi, 1989). There is biological support for this theory, as *J. regia* is considered a relict species of the Tertiary native to the mountains of Xinjiang province in Western China (Renault-Miskovsky et al., 1984; Beer et al., 2008). Starting in the Western Han Dynasty, cultivated walnuts were likely derived from selection of seedlings from geographically distinct natural populations in China and spread by trade and military conquest (Pollegioni et al., 2015; Han et al., 2016; Pollegioni et al., 2017). Today, China is one of the major centers of walnut genetic diversity, serving as a germplasm source for walnut breeding (Chen et al., 2014), and although Persian walnut remains an important crop in China (Chen et al., 2014), the native Chinese common walnut germplasm resource is not well characterized (Gunn et al., 2010; Hu et al., 2014; Wang et al., 2015; Han et al., 2016; Pollegioni et al., 2017).

The aims of this study were as follows: (i) determine the genetic diversity and population structure of *J. regia* in China, (ii) estimate the degree of population differentiation among *J. regia* populations from different regions, and, finally, based on this data (iii) describe the lineage divergence, location(s) of glacial refugia, and phytogeographic history of common walnut in China.

## Materials and Methods

### Sample Collections and DNA Extractions

From 2013 to 2015, leaf samples of 602 *J. regia* individuals were collected from 31 populations across the species’ entire geographical distribution in China (**Supplementary Table S1**). All sampled trees were mature adults, apparently healthy, growing in a mountain forest, along a forest road, or near a village but not in an orchard or on farmed land. Sampled trees were separated by at least 50 m. Fresh leaf samples were stored at 25°C in silica gel until DNA extraction, as previously described (Zhao and Woeste, 2011; Han et al., 2016).

### Genotyping Using Simple Sequence Repeats (SSRs)

We genotyped the DNA samples with 23 EST-SSR markers (**Supplementary Tables S2**, **S3**), four originally from Chinese walnut (*J. cathayensis*) (JC8125, JC7329, JC2995, and JC5411; Dang et al., 2015), four from *J. mandshurica* (JM61666, JM5969, JM68820, and JM78331, Hu et al., 2016), seven from *J. hopeiensis* (JH89978, JH84548, JH42753, JH86514, JH91908, JH2096, and JH6044, Hu et al., 2015), and eight from common walnut, *J. regia* (JR4616, JR3773, JR4964, JR6439, JR1165, JR6160, JR3147, and JR1817, Dang et al., 2016) using PCR and genotyping protocols as previously reported (Dang et al., 2016; Han et al., 2016). The forward primers were labeled with fluorescent dyes, 6-FAM, HEX, TAMRA or ROX from Sangon Biotech in Shanghai, China (**Supplementary Table S2**). We obtained genotypes using the ABI 3730XL sequencing system (Applied Biosystems, Foster City, CA, United States) and analyzed them using GENEMARKER ver. 2.2.0 software (Holland and Parson, 2011).

### Detection of *F*_ST_ Outlier Loci and Population Diversity Statistics

We calculated *F*_ST_ to identify outlier loci potentially under selection (Tsuda et al., 2015). Loci under selection pressure can bias the population genetic analysis because most methods for evaluating genetic structure assume neutrality (Luikart et al., 2003; Antao et al., 2008) (*P* ≤ 0.05; **Supplementary Figure S1**). On the other hand, loci under selection can be used specifically to investigate “population structure on ecological, rather than evolutionary timescales” (Waples and Gaggiotti, 2006; Helyar et al., 2011). After identification of nine loci that showed evidence of non-neutrality (**Supplementary Figure S1** and **Supplementary Table S3**), the remaining 14 markers (including locus JM68820; **Supplementary Table S2**) were again evaluated for *F*_ST_ values and Hardy–Weinberg equilibrium in each population using default parameters in GENEPOP ver. 4.5.1 (Raymond and Rousset, 1995). Loci were also tested for linkage disequilibrium (LD) across all populations using FSTAT ver. 2.9.3 (Goudet, 1995). Frequency of null alleles was predicted using MICRO-CHECKER ver. 2.2.3 (Van Oosterhout et al., 2004). We determined that locus JM68820 had more null alleles than other loci and it was not in Hardy–Weinberg equilibrium, so it was deleted from all subsequent analyses (*P*-value < 0.01) (**Supplementary Table S4**).

The nine loci determined to be *F*_ST_ outliers (likely to be under selection) were used for analyses related to contemporary distribution and population assignment only (**Supplementary Figure S1** and **Supplementary Table S3**). All loci were annotated using Pfam, GO database, and blastx of NCBI. For each population and locus we calculated the number of alleles (*N*_a_), number of effective alleles (*N*_e_), Shannon’s Information index (*I*), observed heterozygosity (*H*_O_), and expected heterozygosity (*H*_E_), and Fixation index (*F*_IS_), Nei’s genetic distance, and the percentage of polymorphic loci (*PPL*) using GENEALEX ver. 6.5.0.1 (Peakall and Smouse, 2006) (**Supplementary Table S1**). The inverse distance weighted (IDW) interpolation function implemented in the GIS software ArcGIS ver. 10.3 (ESRI, Redlands, CA, United States) was used to show the geographic patterns of observed heterozygosity (*H*_O_), number of alleles (*N*_a_), the percentage of polymorphic loci (*PPL*), allelic richness (*R*_s_), and the number of private alleles (*Pr*_A_) was computed for all 31 common walnut populations using ArcGIS ver. 10.3 software.

### Population Differentiation Statistics

To test whether allele frequencies differed among populations or groups, we used GENEPOP ver. 4.5.1 (Raymond and Rousset, 1995), which tests the null hypothesis that population allele frequencies are drawn from the same distribution. The pairwise *F*_ST_ values between each population pair were calculated using FSTAT (Goudet, 1995) and tested for significance using 1,000 permutations. We examined genetic distance between populations using principal coordinate analysis (*PCoA*) of pairwise population differentiation (*F*_ST_), generated with GENALEX (Peakall and Smouse, 2006). Finally, we performed an analysis of molecular variance (AMOVA) based on Bayesian structure analysis of two data sets, one that included loci under selection (22 SSR loci in total, excluding JM68820, which resulted in *K* = 4) and a second analysis that excluded loci under selection (13 loci, *K* = 3) using ARLEQUIN ver. 3.5 (Excoffier and Lischer, 2010).

### Genetic Structure

We performed genetic structure analysis using the software STRUCTURE ver. 2.3.4 (Pritchard et al., 2000) based on 22 SSR loci, or separately, based on 13 neutral SSR loci (leaving out nine non-neutral loci) and 9 non-neutral SSR loci. STRUCTURE software identifies populations that diverged in allele frequency when gene flow was limited or non-existent, presumably because of adaptation to local environmental conditions or as a result of divergent selection pressures. We also performed genetic structure analysis based on the nine non-neutral loci (Cheviron and Brumfield, 2009; Miller et al., 2010; Freamo et al., 2011). For each dataset, a burn-in of 500,000 Markov Chain Monte Carlo iterations was followed by 200,000 iterations and 15 replicates per run for *K* from 1 to 15 clusters and 15 independent runs with the admixture model (Pritchard et al., 2000; Evanno et al., 2005). The program STRUCTURE HARVESTER was used to calculate the optimal value of *K* using the delta *K* criterion (Earl and vonHoldt, 2012), the inferred clusters were drawn as colored box-plots using the program DISTRUCT ver. 1.1 (Rosenberg, 2004).

### Estimates of Demographic History

To decipher the demographic history and postglacial dispersal routes of *J. regia* in China, we estimated divergence times, admixture, and changes in populations size among different populations using the Approximate Bayesian Computation approach (ABC) in DIYABC ver. 2.0.4 (Cornuet et al., 2014) based on the subset of 13 neutral SSR loci. We pooled the Chinese common walnut samples into three groups (populations A, B, and C) as inferred by STRUCTURE analysis based on 13 neutral SSR loci (**Supplementary Figures S2**–**S4** and **Supplementary Tables S1**, **S5**). Population A consisted of eight demes (BM, BZ, DZ, HL, LM, QH, XJ, and XZ) from the Xinjiang Uygur Autonomous Region, Tibet, Yunnan province, and Northern China (Western, Southwestern China, and Northern China); population B consisted of six demes (BS, EM, F, GZ, SC, and YN) from Yunnan province and Sichuan province (Southern China), and population C consisted mostly of seventeen demes (AH, CQ, GS, HN, JJ, LNS, MT, NY, PD, SX, ZS, BJX, HB, HHG, LN, PL, and ZJP) from the Qingling-Bashan Mountains (Central China) (details see **Supplementary Figure S2** and **Supplementary Table S1**).

For each of the three derived populations (A, B, and C), we estimated the following summary statistics: (i), mean number of alleles; (ii), mean genetic diversity; (iii), mean size variance (one sample summary statistics); for each of the population pairs, *F*_ST_, classification index, and (dμ)^2^ distance (Tsuda et al., 2015) (**Supplementary Figure S3** and **Supplementary Table S6**). These statistics were used in 13 scenarios for analysis using DIYABC. In these scenarios, *t*# represented the time scale measured in number of generations, *N*_#_ represented effective population sizes, *N*_A_ represented ancestral population size, and t2 > t1, t3 > t1, t2 < t3. We performed every scenario with 1,000,000 simulations. In concordance with Bai et al. (2016), we assumed a generation time of 50 years. When all simulations were finished, we tested the posterior probability parameters (**Supplementary Figure S4**). In order to identify the best-supported scenario, we estimated posterior distributions using a weighed polychotomous logistic regression value of 1% of simulated posteriors data sets closest to the observed data from posterior probabilities of scenarios (the mean of the closest 1% of simulated posteriors). The goodness of fit of the best supported scenario was evaluated by the option ‘model checking’ with principal component analysis (*PCA*) (**Supplementary Figure S4**). The median value of the mean mutation rate for SSR was 2.14 × 10^-6^ (95% CI: 1.08 × 10^-6^–9.48 × 10^-6^), the mean *P* (the parameter of the genomic distribution to generate multiple stepwise mutations) was 0.626 (95% CI: 0.173–0.9), and Single Nucleotide Indel (SNI) was assessed to be 4.17 × 10^-8^ (1.01 × 10^-8^–1.83 × 10^-6^).

### Chloroplast Haplotype Analysis

**FIGURE 1:**
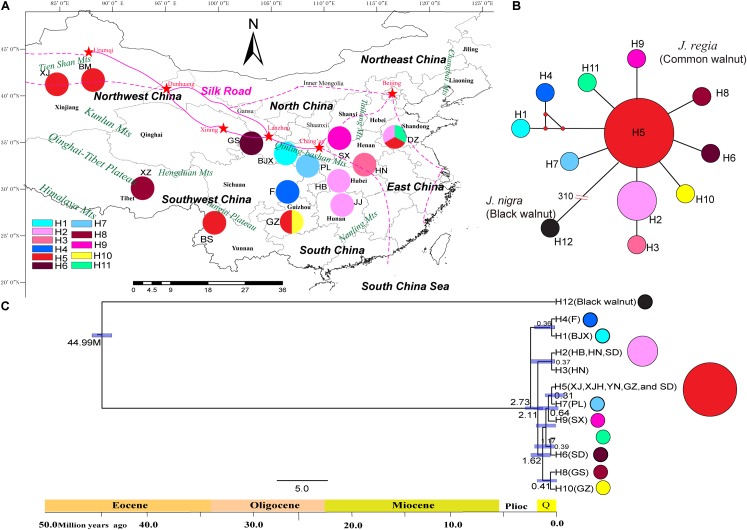
Geographical distribution of haplotypes of 17 individuals of common walnut (*Juglans regia*). **(A)** Geographical distribution of 11 chloroplast haplotypes in *J. regia* and sampled locations. Colors of haplotypes correspond of those of the small figure in the left corner of the map. **(B)** The minimum spanning network of 11 chloroplast genome haplotypes rooted by *J. nigra*. In the network diagram, small red circles indicate intermediate haplotypes not detected in the dataset; red split lines and numbers indicate mutation steps supported by indels, while black lines indicate one mutation step supported by indels. The black circles indicate the haplotype of *J. nigra*. The small red dot indicated that the mutation step. The number “310” on the truncation of a branch of black walnut represent a total of 310 mutation steps. No red dot between haplotypes represent only one mutation step. **(C)** BEAST-derived chronograms of 11 chlorotypes of *J. regia* based on whole genome sequences. Blue bars and the numbers above the bars indicate 95% highest posterior densities (HPDs) of time estimates [million year ago (Mya)].

To understand the demographic history of each maternal lineage of *J. regia* in China, we sequenced and assembled the complete chloroplast genomes of 17 individuals chosen based on results of genetic structure analysis (**Supplementary Table S1**; details of methods see Hu et al., 2017). The 17 individuals were distributed from throughout the Chinese range of common walnut. The chloroplast haplotypes were calculated using DNASP ver. 5 (Librado and Rozas, 2009). The sequence data were used to generate median-joining (MJ) haplotype networks (**Figure 1**). The divergence time for the major lineages were estimated as implemented in BEAST ver. 2.3.2 (Bouckaert et al., 2014). Fossil data within *Juglans* (divergence of black walnut, *J. nigra*, 38 ∼ 45 Mya) and a mutation rate 10 × 10^-8^ were used to estimate divergence time (Manchester and Garden, 1987; Bai et al., 2016).

### Species Distribution Modeling

Species distribution records were retrieved from the Chinese Virtual Herbarium^1^ and National Specimen Information Infrastructure^2^. We obtained about 2,750 records. There were many redundant records, however, so we chose 284 *J. regia* records to generate a species distribution model (SDM) for *J. regia* based on maximum entropy modeling using MAXENT ver. 3.3.3 (Phillips et al., 2006; Phillips and Dudík, 2008).

To explore the potential distribution of *J. regia* under current climatic conditions and predict where suitable conditions were present in the past based on paleoclimate environmental layers, we used 19 biologically meaningful climate variables (BIO1-19, **Supplementary Table S7**). We analyzed four time frames: current (1965–1978, resolution: 2.5 arc-minutes), during the last glacial maximum (LGM) [∼21,000 years (kya), resolution: 2.5 arc-minutes], during the last interglacial (LIG) (120–140 kya, resolution: 30 arc-minutes), and future climate environmental layers (∼2050, resolution: 2.5 arc-minutes) based on data from the Worldclim database^3^. The biologically relevant climate variables of the LGM were accepted from the community climate system (CCSM) and the Interdisciplinary Research on Climate model (MIROC). To identify a best fit model, we defined two datasets: one consisted of the 19 bioclimate layers used to simulate the current distribution of the species; a second used 15 bioclimate layers selected based on the bioclimate variable analyses. To reduce the potential impact of correlation among different bioclimatic variables (Kozak et al., 2008; Wenger and Olden, 2012), we retained variables only if their Pearson’s correlation coefficient with other variables was lower than 0.85 (Cornille et al., 2013). Model evolution statistics were produced from ten bootstrap model runs. The area under the Receiver Operating Characteristic curve (AUC) value was used to assess model performance. Usually, an AUC value from 0.5 to 1.0 indicates a good fit for a random prediction (Anderson et al., 2006; Phillips et al., 2006). Data related to the collection sites of the 31 populations of *J. regia* and the two climate data sets were standardized (with respect to measurement units) and analyzed using “R” software (R Core Team, 2016). In order to examine the relative importance of the climate variables on the species distribution, we evaluated percent contribution, permutation importance, and jackknife tests (Ornelas et al., 2016).

To assess the degree of ecological niche overlap among different genetic groups of *J. regia*, we performed pairwise analyses, examining the niche space between different genetic groups of common walnut as determined by STRUCTURE and ABC. Overlap was evaluated between population A/population B, population A/population C, and population B/population C (see **Supplementary Table S1** for more details). Overlap among niche models was assessed using Schoener’s (1968) and Hellinger’s *I*, calculated in ENMTOOLS ver.1.4.1 (Warren et al., 2010). These metrics range from 0 to 1, indicating no niche overlap and complete niche overlap, respectively (Warren et al., 2008). To determine if the calculated niche similarity metrics were significant, we performed identity tests in ENMTOOLS. We plotted the results of this test using the ggplot2 package (Wickham, 2009) in R (R Core Team, 2016) and assessed significance with a one-sided Wilcoxon test.

### Impact of Environmental Factors on Genetic Structure (Isolation by Environment)

In order to evaluate the effect of present climatic conditions on the observed pattern of genetic differentiation, we tested for the relationship between pairwise genetic differentiation *F*_ST_ (Goudet, 1995) and climatic distance while controlling for geographic distance among the 31 sampled populations. Nineteen bioclimatic variables were determined for the current climate layers as used earlier in ecological niche modeling (ENM). These were summarized into the first two axes of a principal coordinate analyses (*PCoA*) using R ver. 3.1.0. We computed climatic (Euclidian) distance matrices based on population scores for both *PCoA* axes (PC1 and PC2), and for each bioclimatic variable. Tests were performed for the whole data set, including both northern and southern lineages, using partial Mantel tests (‘mantel.partial’ function; R Core Team, 2016) based on 10, 000 permutations.

### Landscape Genetics

The biogeographic boundaries between population pairs were calculated by the Monmonier’s maximum-difference algorithm in BARRIER ver. 2.2 (Manni et al., 2004) based on the multiple distance matrix. Permutation and bootstrap tests were conducted with 1,000 replicates in each case.

### Gene Flow Analysis

We used MCMC maximum likelihood (ML) to estimate historical gene flow among the populations A, B, and C, and among populations I, II, III, and IV using MIGRATE ver. 3.6 (Beerli and Felsenstein, 2001). Population by code “A,” “B,” and “C” indicate metapopulations used in the DIY-ABC analysis as identified using STRUCTURE based on 13 neutral SSR loci (**Supplementary Figure S2**). Population “I,” “II,” “III,” and “IV” indicate metapopulations as identified using STRUCTURE analysis based on all 22 SSR loci (13 neutral and 9 non-neutral) (for details concerning metapopulations, see **Supplementary Table S1**). Based on EST-SSR data, the parameters θ (four times effective population size multiplied by mutation rate per site per generation) and M (immigration rate divided by the mutation rate) were calculated using software MIGRATE ver. 3.6. First, we implemented *F*_ST_ estimations for the parameters θ and M. Then we started four independent runs by 10 short chains of 5,000 steps and 3 long chains of 50,000 steps. We recorded genealogies with a sampling increment of 100 and 10,000 burn-in records. Last, we calculated the average values as the true value. To estimate contemporary gene flow among the meta-populations A, B, and C (I, II, III, and IV), we used BAYESASS ver. 3.0 (Wilson and Rannala, 2003) to calculate migration rates, inbreeding coefficients, and allele frequencies.

## Results

### Genetic Diversity of *J. regia* in China Based on EST-SSRs

Among the 22EST-SSR loci we examined, nine showed evidence of positive selection with 95% confidence intervals (CI) based on *F*_ST_-outliers (JM5969, JR4964, JH84548, JR6160, JH6044, JR3147, JH91908, JR1165, and JR1817) when a false discovery rate (FDR) was considered (**Supplementary Figure S1**). The 13 EST-SSRs that were not under selection were assumed to be neutral SSRs (**Supplementary Figure S1**). Eighteen loci out of the 22 polymorphic EST-SSRs showed significant departures from Hardy–Weinberg equilibrium (HWE) across all samples (**Supplementary Table S8**).

A total of 602 individuals were successfully genotyped. We observed an average of 6.79 alleles per each of 22 SSR loci for a total of 95 alleles in 31 *J. regia* demes (**Supplementary Tables S2**, **S3**). The number of alleles (*N*_a_) for each locus ranged from 2 to 19 (**Supplementary Table S2**). Eight of the 22 loci (34.8%; JH89978, JC7329, JM61666, JC2995, JC5411, JM78331, JH2096, and JR6494) were monomorphic in more than 10 of the 31 populations. The observed heterozygosity (*H*_O_) and expected heterozygosity (*H*_E_) varied from 0.13 to 0.40 (-*x* = 0.23) and from 0.08 to 0.50 (-*x* = 0.30), respectively (**Supplementary Table S1**). The percentage of polymorphic loci per population (*PPL*) ranged from 26.1 to 95.7%, with a mean of 65.8%.

**FIGURE 2:**
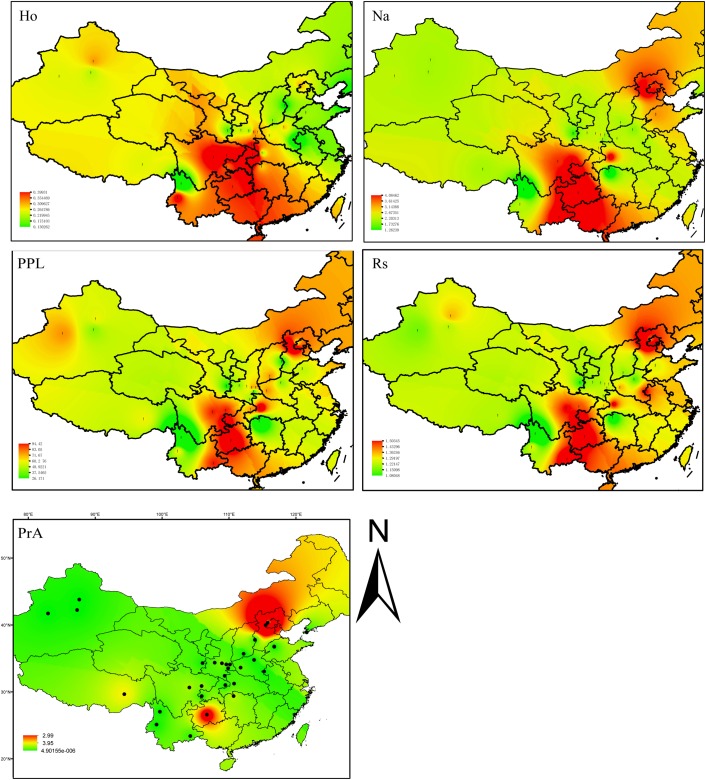
Inverse distance weighted (IDW) interpolation of the observed heterozygosity (*H*_O_), number of alleles (*N*_a_), percentage of polymorphic loci (*PPL*), allelic richness (*R*_s_), and the number of private alleles calculated for 31commonwalnut populations (black dots) in the species, China range using 22 EST-SSR markers. The numbers of walnut populations are shown in **Supplementary Table S1**.

Genetic diversity appeared to be highest in two distinct locations (Southwestern China, including BS, YN, GZ, CQ, EM, and SC; and Northern China including HL, MT, and LNS). Trees sampled from these demes were especially genetically diverse as measured by number of alleles (*N*_a_), percentage of polymorphic loci (*PPL*), and allelic richness (*R*_S_), although only the populations from Southwestern China showed high observed heterozygosity (*H*_O_) values (**Figure 2** and **Supplementary Table S1**). The sampled site with the highest diversity was MT based on *PPL* (95.65%), I (0.89), and *H*_O_ (0.21). The site with the lowest diversity based on these measures was LM (near the Tibet/Yunan border) (*PPL* = 26.09%, *I* = 0.12, *H*_O_ = 0.13) (**Figure 2** and **Supplementary Table S1**).

Of 190 alleles detected, 56 were unique to a single geographic site. The samples from MT had 21 private alleles at 13 loci, while GZ had 11 private alleles at 10 loci (**Figure 2** and **Supplementary Tables S9**, **S10**). Private alleles at the GZ sample site ranged in frequency from 0.028 to 0.227; in MT, private allele frequency ranged from 0.033 to 0.335; in HL at JH91908 four private alleles were observed at a frequency of 0.031, 0.031, 0.045, and 0.094. Fourteen sampled sites had at least one private allele at a total of 22 different loci (**Figure 2** and **Supplementary Tables S9**, **S10**).

### Reconstruction of the Past Distribution Shifts of *J. regia*

**FIGURE 3:**
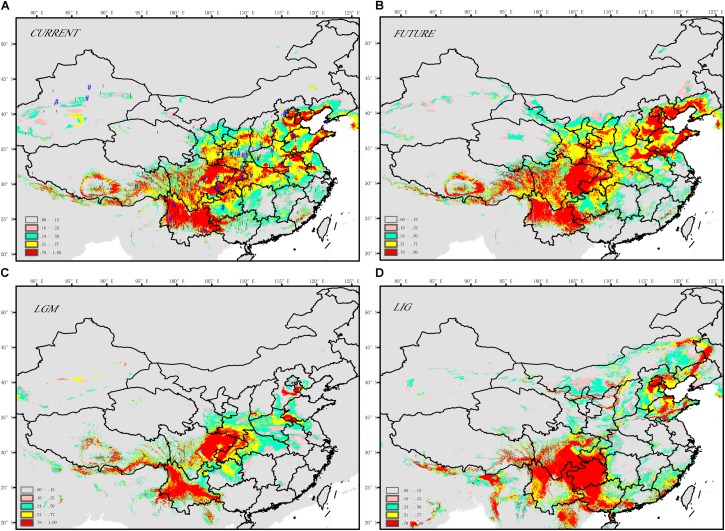
Maps displaying range predictions for *J. regia* based on a species distribution model **(A)** current, **(B)** future ∼ 2050s, **(C)** LGM ∼ 21 ka, **(D)** LIG ∼ 120–140 ka.

The SDM model yielded high AUC scores (AUC = 0.993) and thus was expected to perform well for *J. regia* (**Figure 3** and **Supplementary Table S7**). The current potential distribution as predicted by the model was highly consistent with the actual species distribution. During the LIG, the range of *J. regia* contracted considerably, and the species was probably restricted to parts of Yunnan (Southwestern China), the Southern Qinling-Bashan Mountains, Beijing, Shandong, and Changbai Mountains (Northeastern China) (**Figure 3D**). Later, during the LGM, our models show a general southward range shift; although the species did not disappear from the northeastern part of its range, it was likely limited to a small region (near present-day Beijing) (**Figure 3C**). The distribution map reconstructed from the SDM model showed that the occurrence of *J. regia* from the LIG and into the future was/will be highest in Southwest China (Yunnan, Sichuan, and Guizhou provinces) and Northeast China (Liaoning, Jilin, and Heilongjiang provinces) (Prob. >0.75). Accordingly, we predicted a gain of suitable habitat in Northeast China (e.g., Changbai Mountains). The species will be most common in Northeast China in the future based on the SDM model (**Figure 3**).

According to the relative importance tests, BIO9 (**Supplementary Table S7**; mean temperature of the driest quarter) was the most important layer for determining the distribution of *J. regia*. Subsequent layers, including BIO18 (precipitation of warmest quarter), BIO11 (mean temperature of coldest quarter), and BIO15 (precipitation seasonality, coefficient of variation) also affected the species distribution. The four most important ecological factors were precipitation of the warmest quarter (16.9%), mean temperature of the driest quarter (21.6%), precipitation seasonality (coefficient of variation) (15.1%), and mean temperature of the coldest quarter (10.4%) (**Supplementary Table S7**).

### Inference of Population Demographic History of Walnut in China

**FIGURE 4:**
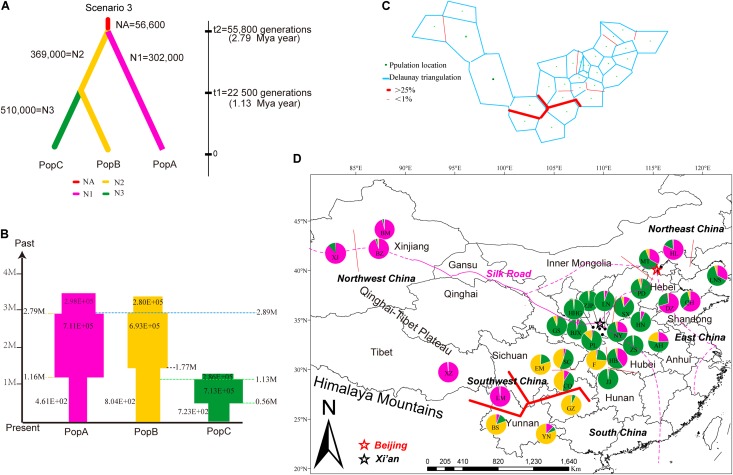
Summary of inferred demographic history of the three genetic clusters of *J. regia*. **(A)** The three scenarios tested in DIYABC. In these scenarios, *t*# represents time scale in terms of the number of generations and *N*# represents the effective population size during the time period (e.g., 0–t1, t1–t2), and NA = ancestral. The summary shows the estimated demographic parameters of the most likely scenario, number 3, in DIYABC. **(B)** Changes in population size and their timing are indicated by the width of each cylinder. Times of divergence and changes in population sizes are indicated by horizontal dashed lines. **(C)** Results of the BARRIER analysis based on microsatellite data, showing the spatial separation of *J. regia* populations. Delaunay triangulation and detected barrier (thick red line) separating LM, Tibetan, and Xinjiang demes from other demes in Southwest China. Bootstrap values over 1000 replicates using Nei’s genetic distances (1983). **(D)** Locations of the 31 sampled demes of *J. regia* and their color-coded grouping at the most likely *K* = 3 based on 13 neutral SSR markers (**Supplementary Figure S5**). Geographic location of the genetic barrier **(C)** is indicated by a red line.

Applying Bayesian analysis of genetic structure to the trees from all 31 sampled sites using only the 13 neutral loci, the most likely number of populations was *K* = 3 (**Supplementary Figure S2**). Based on these three metapopulations (populations A, B, and C), we evaluated 13 scenarios related to the phylogeny and demography of *J. regia* in China (**Figure 4**, **Supplementary Figure S3** and **Supplementary Table S3**). DIYABC unambiguously indicated support for scenario 3 (0.2770, 95% CI: 0.2527–0.3013), the 95% confidence intervals of this model did not overlap with the 12 other scenarios (**Figure 4A**, **Table 1** and **Supplementary Figure S3**). In all 13 scenarios, there was a significant difference between the observed and simulated data based on posterior distributions of scenarios. The PCA of posterior distributions of scenarios showed that the observed data was always near the cluster of simulated data, indicating that scenario 3 was generally suitable for the observed data (**Supplementary Figure S4**). Scenario 3 posited population A at t2 (2.79 million years ago, 95% CI: 0.68–4.87 Mya BP, during the Pliocene), and a more recent separation of population B and population C at t1 (1.13 million years ago, 95% CI: 0.14–2.33 Mya BP, Pleistocene, in the Quaternary) (**Figure 4A** and **Supplementary Figure S3**).

**Table 1:** Posterior probability of each DIYABC scenario and its 95% confidence interval based on a logistic estimate.

Scenario	Posterior probability	95% CI (lower–upper)
3	0.28	0.25–0.30
5	0.22	0.20–0.24
1	0.18	0.15–0.21


In scenario 3, the median values of the effective population size (*N*_E_) of the ancestral population (*N*_E_^a^) was 56,600 (95% CI: 2,580–572,000), the effective population size of population A (*N*_E_^1^), and population B (*N*_E_^2^), population C (*N*_E_^3^) were 302,000 (95% CI: 66,700–881,000), 369,000 (95% CI: 96,100–896,000), and 510,000 (95% CI: 142,000–952,000), respectively (**Figure 4A**). The size of all three populations has undergone cyclical shrinkage and expansion during the Quaternary (**Figure 4B**). Population A, B, C started to expand their distribution *c.*2.79 Mya, 2.89 Mya, and 1.13 Mya, respectively. At the time of the divergence of population C, the sizes of populations A, B decreased considerably while population C expanded (**Figure 4B**).

We identified 11 chloroplast haplotypes among the 17 *J. regia* individuals we sequenced. Haplotype H5 was widespread and most common, being found in five demes, distributed in Northwest China (XJ and XJH), Southwest China (YN and GZ), and East China (SD) (**Figure 1**). The outgroup *J. nigra* (black walnut) contained a private haplotype (H12) (**Figure 1**). The minimum spanning network of 11 chloroplast genome haplotypes showed that H5 was the ancestral haplotype, but the conclusion that H5 was ancestral was not supported by the BEAST tree (**Figure 1**). Most of the other *J. regia* chloroplast haplotypes were derived by a single mutation from H5. The coalescence time for the 11 chlorotypes was 2.73 Mya (95% HPD: 0.38–5.18 Mya) (**Figure 1C**), dating to the Plio-Pleistocene. The microsatellite data supported diversification of *J. regia* about 2.79 Mya (**Figure 4A**) based on neutral loci. Most chlorotypes diverged during the Quaternary glacial period (**Figure 1C**).

### Spatial Genetic Structure of Common Walnut Populations in China

**FIGURE 5:**
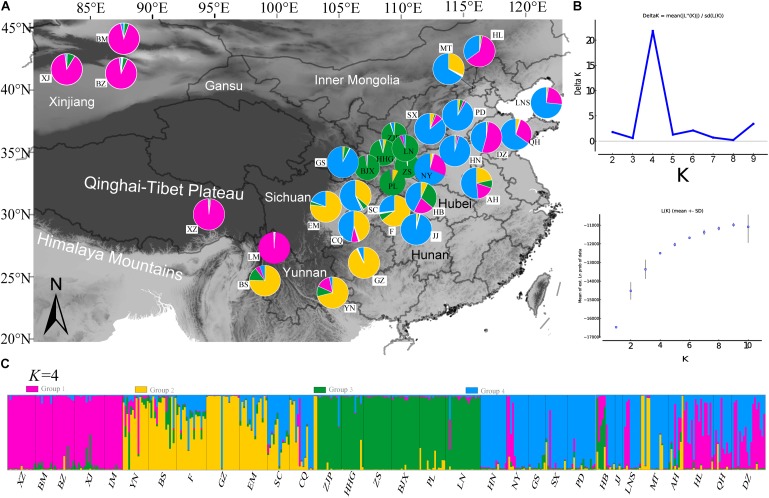
Population genetic structure results of 31 demes of *J. regia* based on 22 SSR loci (neutral and non-neutral). **(A)** Locations of the 31 demes of *J. regia* and their color-coded grouping at the most likely *K* = 4 (**Supplementary Table S1**). Purple, population I; Yellow, population II; Blue, population III; Green, population IV. **(B)** The mean posterior probability value of structure results. **(C)** Histogram showing the proportion of membership in each of the four genetic clusters for each individual in the 31 *J. regia* demes for the inferred clusters when *K* = 4 according to STRUCTURE analysis.

The STRUCTURE analysis based on 22 loci (13 neutral microsatellites and 9 non-neutral loci) indicated *K* = 4 was the best representation of the underlying hierarchical structure for the 31 common walnut demes we sampled (the highest Δ*K* value was detected to *K* = 4, **Figure 5** and **Supplementary Table S1**). Most of the samples clustered into predicted populations that corresponded to the geographic region from which they were sampled (**Figure 5A**). For *K* = 4, eight populations from Xinjiang (XZ, BM, and BZ, Northwestern China), two populations (XZ and LM) from Tibet and Yunnan (Southwestern China), and populations from Hebei and Shandong provinces (HL, QH, and DZ, Eastern China) clustered into population I (**Figure 5** and **Supplementary Table S1**); samples from seven demes from Yunnan and Sichuan province (Yunnan-Kweichow Plateau region, Southwestern China, **Figure 5** and **Supplementary Table S1**) clustered into population II (YN, BS, GZ, F, EM, SC, and CQ; **Supplementary Table S1**); samples from nine demes from Northern China clustered into genetic population III (GS, JJ, HN, PD, NY, SX, LNS, MT, and AH), and samples from six demes from Qinling-Bashan Mountains were clustered into genetic population IV (HHG, ZJP, BJX, PL, LN, and HB; **Figure 5** and **Supplementary Table S1**). Populations MT, LNS, AH, SX, NY, and HB contained members from population I (purple) and population (blue), while CQ, SC, and AH were mostly comprised of population II (yellow) and population III (blue) (**Figure 5**). Principal coordinate analyses (*PCoA*) accounted for 54.82% of the observed variance on the first two coordinate axes (**Supplementary Figure S5**) and revealed four clusters of samples corresponding to the same groups identified by STRUCTURE (**Supplementary Figure S5**).

AMOVA analyses showed that genetic differentiation (*F*_ST_) was also high (0.15) among the four subpopulation I, II, III, and IV based on the 22 loci (**Table 2**). The matrix of *F*_ST_ values between the 31 demes (**Supplementary Table S11**) and the matrix of *F*_ST_ values between the four populations (as determined by STRUCTURE) showed that population I and population II had the highest genetic differentiation (*F*_ST_ = 0.148). The highest *F*_ST_ (0.544) between two demes was found between JJ and LM (**Supplementary Table S11**).

**Table 2:** Results of AMOVA based on 22 EST-SSR^a^.

Sets	Source of variation	d.f	Sum of squares	Variance components	Percentage of variation	Fixation index
All pops	Among populations	30	345.19	0.39Va	29.08	*F*_ST_ = 0.29
	Among individuals within populations	377	448.98	0.23Vb	17.28	*F*_IS_ = 0.25
	Within individuals	408	295.5	0.72Vc	53.64	*F*_IT_ = 0.46
	Total	815	1089.67	1.35		
Four pops	Among groups	3.00	658.59	0.94Va	15.40	*F*_CT_ = 0.15
	Among populations within groups	27.00	717.39	0.78Vb	12.75	*F*_SC_ = 0.15
	Among individuals within populations	377.00	2367.27	1.89Vc	30.89	*F*_IS_ = 0.43
	Within individuals	408.00	1021.50	2.50Vd	40.92	*F*_IT_ = 0.59
	Total	815.00	4764.74	6.11		


**^*a*^Includes both neutral and non-neutral loci*.*

### IBD and Landscape Effect on Walnut Population Structure

The Mantel test for matrix correlation revealed a strong and significant correlation between genetic distance and geographic distance (*r* = 0.5608, *P* < 0.001; **Supplementary Figure S6**). Analyses using the software BARRIER identified five statistically significant (0.25 < *P* < 0.41) genetic barriers (**Figures 4C,D**). The main genetic boundary appeared between LM (purple demes in **Figure 4**) and BS (yellow demes in **Figure 4**) corresponding to a barrier between the Himalayan samples and those of Yunan (Yunnan-Kweichow Plateau region, Southwestern China). Within the demes from Southwest China a second genetic barrier appeared (**Figures 4C,D**). Haplotype H6 was private to Tibet (XZ); most members of population I, including samples from Xinjiang (XJ and BM) and Yunnan (BS) contained H5 (**Figure 1**). Estimates of θ were lowest for population I (**Table 3** and **Figure 5**). Scaled immigration rates (*M*) revealed asymmetric historical gene flow from population I to population II (*M* = 5.14), population III (*M* = 0.92), and population IV (*M* = 0.53) (**Table 3**). The scaled immigration rate (*M*) was 15.8 from population B (yellow, **Figure 4**) to population C (green, **Figure 4**), while immigration rate (*M*) was 1.5 from population B to population A (purple, **Figure 4**). These results revealed the existence of generally asymmetric historical gene flow among metapopulations. Gene movement occurred mainly from population B into population C (15.8 vs. 11.9) and from population I into population II (5.14 vs. 3.81) (**Table 3**).

**Table 3:** Estimated gene flow among *J. regia* populations.

		*M*(m/μ)					
		
	θ	Pop I→	Pop II→	Pop III→	Pop IV→
Pop I	0.31 (0.27–0.35)		3.81 (3.56–4.06)	2.86 (2.66–3.08)	2.24 (2.06–2.43)
Pop II	0.50 (0.49–0.51)	5.14 (4.88–5.42)		0.95 (0.84–1.08)	1.07 (0.95–1.20)
Pop III	0.59 (0.58–0.60)	0.92 (0.84–1.00)	1.16 (1.07–1.26)		0.75 (0.67–0.83)
Pop IV	0.57 (0.55–0.58)	0.53 (0.47–0.60)	0.86 (0.78–0.94)	0.96 (0.88–1.05)	

		***M*(m/μ)**					
		
	**θ**	**PopA→**	**PopB→**	**PopC→**

Pop A	2.3 (2.3–2.4)		1.5 (1.3–1.4)	2.1 (1.9–2.2)
Pop B	0.5 (0.5–0.6)	7.4 (7.0–8.0)		11.9 (11.2–12.6)
Pop C	1.9 (1.9–2.0)	9.6 (9.2–10.0)	15.8 (15.2–16.4)	


**Population code (pop), “I,” “II,” “III,” and “IV” refer to genetic clusters as determined by Bayesian analysis using software STRUCTURE (Pritchard et al., 2000) based on 22 SSR loci. Population codes, “A,” “B,” and “C” refer to genetic clusters as determined by Bayesian analysis using software STRUCTURE (Pritchard et al., 2000) based on 13 neutral SSR loci; details see **Supplementary Table S1**. θ = four times effective population size multiplied by mutation rate per site per generation M = immigration rate divided by the mutation rate. The median value of the mean mutation rate for SSR was 2.14 × 10^-6^ (95% CI: 1.08 × 10^-6^–9.48 × 10^-6^) (Tsuda et al., 2015; Bai et al., 2016)*.*

**FIGURE 6:**
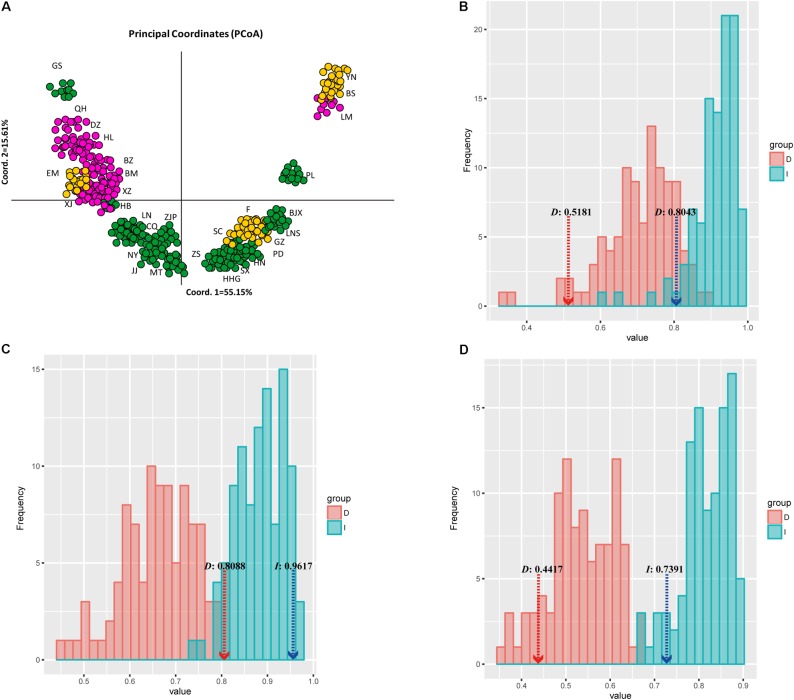
Plot of sampled sites or occurrence data for each of common walnut *J. regia* onto two principal components that summarize 70.76% of variance in 19 bioclimatic layers. Each taxon is represented by a different color, as indicated in the box at lower right of **(A)**; **(B)** Niche identity tests for population A of *J. regia*/population B of *J. regia*, **(C)** Niche identity test for population A of *J. regia*/population C of *J. regia*, and **(D)** Niche identity test of population B of *J. regia*/population C of *J. regia*. Light red indicates Schoener’s *D* and its null distributions of 1,000 pseudo replicates; light blue indicates Warren’s et al. (2010). I and its null distributions of 1,000 pseudoreplicates. The dashed lines denote the observed values of Schoener’s *D* and Hellinger’s *I* with significant *P*-values for both niche overlap measures (*P* < 0.001).

The *PCoA* of ecological niche showed significant ecological divergence between populations A, B, and C (see **Figure 4**) predicted by STRUCTURE analysis of the *J. regia* samples (**Figure 6A**) based on neutral loci only. Although many geographically proximal demes were also proximal in the *PCoA* space, there were notable exceptions, and apparent genetic similarity was not always reflected in similarity of ecological niche. For example demes GS (green), EM (yellow), and LM (purple) all deviated sharply from their predicted niches (**Figure 6A** and **Supplementary Table S1**). The genetic barrier between LM and BS (**Figure 4D**) was not reflected in the niche *PCoA*. *J. regia* is adapted to a surprisingly large range of conditions (**Figures 3**, **6**).

The loadings of the *PCoA* analysis showed that interactions of temperature and precipitation were the most important factors driving the distribution of *J. regia*. The ranges of the three predicted populations (population A, B, and C, **Figures 4**, **6**) of common walnut overlap, but they may not share niches within the same locations (e.g., subpopulation A and subpopulation B; **Figure 6B**). Inspection of the spatial overlap between Ecological Niche Models (ENMs) (**Figure 6**) revealed that factors other than those described in the ENM may maintain parapatry for *J. regia* genetic sub-groups.

## Discussion

### Genetic Diversity of *Juglans regia* in China

Genetic diversity of *J. regia* in China was comparable to values reported for wild populations throughout the species’ range at EST-SSR markers. We found an average of 6.79 alleles per locus at 22 EST-SSR loci, a value lower than reported in some recent studies of *J. regia* based on nuclear SSRs. For example, Aradhya et al. (2017) found a mean of 12 alleles per locus for *J. regia* distributed in five major regions (Gaucasus, Central Asia, East Asia, Southwest Asia, and Eastern Europe) based on 643 genotypes comprising 317 diverse accessions at the National Clonal Germplasm Repository, USDA-ARS, Davis, California, and Pollegioni et al. (2017) observed a value of 14.2 for the 91 common walnut populations in Europe they studied. In general, however, *N*_a_ in our study was similar to values described by Gunn et al. (2010) (*N*_a_ = 7.5), Pollegioni et al. (2011) (a range of 3 to 14, mean was 6.2), Ebrahimi et al. (2016) (a range of 2.7 to 5.6, mean was 4.7); and Aradhya et al. (2017) (7 to 20, mean was 12) for nrSSRs. Observed heterozygosity and expected heterozygosity (*H*_O_ and *H*_E_) are common measures of genetic diversity (Slatkin and Barton, 1989). In our study, the heterozygosity of *J. regia* in China was lower than that reported for other *Juglans* species at both of EST-SSRs and nuclear SSRs (Bai et al., 2014; Laricchia et al., 2015; Hu et al., 2016), and even lower than previously reported for *J. regia* in China based on EST-SSRs (Han et al., 2016). The average *H*_O_ (confidence interval) was 0.21 (0.00–0.65), 0.29 (0.00–0.64), 0.24 (0.00–0.59), 0.20 (0.00–0.54) in populations I, II, III, and IV (population structure based on all 22 loci), respectively (**Table 2**). By comparison, *H*_O_ in *J. regia* sampled across Eurasia was about 0.5 (Aradhya et al., 2017), and *H*_O_ was 0.559 in 91 common walnut populations from the Middle East, Western and Central Asia (Pollegioni et al., 2017) based on nuclear microsatellites (nrSSRs). The range and mean values for *H*_O_ observed in other recent studies ranged from 0.343 to 0.722 (mean was 0.62; Gunn et al., 2010), 0.447 to 0.760 (mean was 0.64; Pollegioni et al., 2011), 0.470 to 0.750 (mean was 0.67; Ebrahimi et al., 2016), and 0.422 to 0.864 (mean was 0.70; Aradhya et al., 2017) based on nrSSRs. In our study, the values of *H*_O_ were lower than *H*_E_ at all 22 loci, revealing a deficiency of heterozygotes compared to theoretical expectations. A deficiency of heterozygotes of this type may be the result of inbreeding which, in our samples could have been the result of local and/or range wide forces including a bottleneck, assortative mating, and small population sizes (**Supplementary Table S1**). Our results may have differed from others in part because we used EST-SSRs, which often have lower levels of polymorphism than nuclear microsatellites that are not located in genes, and therefore EST-SSRs reveal lower heterozygosity than SSR loci in intergenic regions. We also observed numerous demes that were fixed for a single allele at a locus (**Supplementary Table S8**).

The AMOVA showed that *F*_ST_ among populations (or, alternatively, among groups and among populations within groups) was surprisingly high (∼0.30). We also observed a surprisingly large number of private alleles, especially in MT and GZ, possibly reflecting genetic isolation (**Supplementary Tables S9**, **S10** and **Figures 2**, **4C,D**). IBD was significant for *J. regia* in China (**Supplementary Figure S6**), so geographical distance likely restricted gene flow and increased genetic differentiation (reflected in high *F*_ST_ values). In general, it appears that forces such as isolation and adaptation that tend to increase genetic differentiation of local demes have been stronger than homogenizing forces such as gene flow (**Figures 3–5** and **Supplementary Figures S2**, **S6**, **S7**). Local gene flow dynamics among wild demes of *J. regia* in Asia are not well studied, although there is some evidence for landscape scale gene flow processes among Chinese *Juglans* species (Bai et al., 2010). The presence of gene flow in forest trees does not exclude genetic differentiation and adaptation to local and regional environments (Savolainen et al., 2007), but information about the effects of scale (dimensions) and geography on *J. regia* diversity is lacking. Hu et al. (2014), who studied the diversity of *J. regia* in the Qinling mountains based on ITS sequence data, found that genetic variation was mainly within populations (53.40%) with low genetic differentiation among 31 collection sites (Hu et al., 2014). Factors that produce or mitigate isolation of *J. regia* populations include geography (e.g., mountain ranges) (Pollegioni et al., 2014), human dispersal (Pollegioni et al., 2015), climate (mean temperature of driest quarter, and precipitation of warmest quarter), the strength and direction of prevailing winds (Bai et al., 2010), and even the absence of megafauna capable of long-distance dispersal (Turvey et al., 2013).

Our analyses of contemporary gene flow (using BAYESASS) among (inferred) populations were based on neutral SSRs only. The values of contemporary gene flow ranged from 0.0069 to 0.0752 (**Supplementary Figure S8**). These indicated that gene flow from population A (Western China and Northern China) into population C (Central China) (**Supplementary Figure S8**) was greater than gene flow from population B (Southern China) into population C (0.0752 versus 0.0614). This result may reflect the relative strength of monsoonal air flow in the spring when *J. regia* blooms (Bai et al., 2010, 2014). Gene flow was very weak between population A (Western China and Northern China) and population B (Southern China). Geographic and climatic factors probably contribute to this barrier (including mountains, rivers, mean temperature of the driest quarter, and precipitation of warmest quarter) (**Figure 4D** and **Supplementary Table S7**). These climate factors correspond to temperate versus subtropical climates at the sampling sites (**Figures 1**, **4**). The southwesterly migration of different regions before 1.13 Mya is probably affected by one factor: natural gene flow, which was shown by the results of the analyses using MIGRATE software. Population C is predicted to have large amounts of bidirectional gene flow with population B (15.8 and 11.9, **Table 3**; Du et al., 2017). Evidence that contemporary populations of walnut in Southern and Central China receive gene flow from population (population A) in the far Southwest China (**Figure 4** and **Supplementary Figure S8**), from the Himalayas, and (possibly) from other species, increases the likelihood that this type of natural gene flow occurred over the past 2 Mya, shaping the diversity of walnut in China.

It is not clear from our data to which extent isolation and ecological specialization have contributed to genetic differentiation of *J. regia* in China. Han et al. (2016) suggested that Southwestern China, Qinling-Daba Mountains, and Northeastern China should be regarded as high priority areas for conservation of *J. regia* because of the rich genetic diversity found there (Han et al., 2016). Our results indicated that Southwestern China and Northeastern China harbored the highest number of alleles, heterozygosity, and frequency of private allele (**Figure 2**). Although we found a large number of private alleles in MT, this population did not present the highest average pairwise *F*_ST_, so the presence of private alleles may not indicate genetic isolation. The niche *PCoA* did not put MT and GZ in extreme niches, so the private alleles they maintain are likely not the result of adaptation to a specific environment. Although we did not determine chloroplast haplotypes for any samples from MT, some GZ trees had a rare chloroplast haplotype (H10). It is possible that these demes are not genetically or ecologically isolated but actually reflect gene flow into *J. regia* from *J. cathayensis* or *J. mandshurica*. Hybrids are often fertile in *Juglans* (Pollegioni et al., 2009; Zhao and Woeste, 2011; Crystal et al., 2016; Zhao et al., 2018). Although we could not conclude what forces contributed to differentiation of *J. regia* in China, our results increase knowledge of the population structure of *J. regia* in China, indicate the geographic location of different gene pools, and could help rationalize and prioritize reservoirs of genetic diversity (**Figure 4**).

### The Origin of Common Walnut in China

Common walnut is considered a relict species of the Tertiary (Renault-Miskovsky et al., 1984) and native to the mountain ranges of Central Asia extending from Xinjiang province of Western China, parts of Kazakhstan, Uzbekistan, and Southern Kyrgyzstan in Central Asia, and the mountains of Nepal, Tibet, Northern India, and Pakistan, west through Afghanistan, Turkmenia, and Iran to portions of Azerbaijan, Armenia, Georgia, and Eastern Turkey (McGranahan and Leslie, 1991). During the Pleistocene, differentiation of populations may have been influenced by the repeated extinction and colonization during the Quaternary climate oscillations (**Figures 1**, **3**, **4**). The effects of Quaternary climatic oscillations on the distribution and phylogeographic structure of species in the mid- to high-latitude regions of Europe and North America (Hewitt, 2004; Emerson et al., 2010; Ren et al., 2017), in high-altitude areas (Qiu et al., 2011; Liu et al., 2014; Wen et al., 2014; Sun et al., 2015), and Northern China (Chen et al., 2008; Tian et al., 2009; Bai et al., 2010; Zeng et al., 2015) have already been described. However, few studies have examined the biogeographic history of species native to both temperate and subtropical China (Bai et al., 2014), and our analysis therefore provided an opportunity to uncover the detailed Quaternary demographic history of a widely distributed, economically important crop, and to better understand the processes playing a role in their distribution in China. The first divergence between the northwestern and other regional populations of *J. regia* (**Figure 4**) was likely about 2.79 million years ago (Pliocene of the Tertiary), although a more recent date for speciation within *Juglans* has been proposed (Bai et al., 2017). In China, our models predicted that during the late Pleistocene to Pliocene, common walnut (*J. regia*) was distributed in Northeast China, Western Yunnan province, and probably Xinjiang province, based on a species distribution model (**Figures 3**, **4**), chloroplast data (**Figure 1**), and the current genetic structure of common walnut in China based nuclear SSRs (**Figures 3**, **4**).

During LIG, the predicted habitat for *J. regia* in China was split between a northern and a southern range (**Figure 3D**). By the LGM, refugia corresponding to three modern regional centers of genetic diversity were present (**Figures 3C**, **5A**), including locations in Northern, Central, and Southern China. These results showed that intraspecific divergence in *J. regia* was partly driven by climate change. The size of at least one population (A) of this species experienced slight bottlenecks during the last glaciations (**Figures 3**, **4B**). A possible recent expansion of population size in China detected by SDM was also supported by our genetic data (**Figures 3**, **4**). The expansion to Northern China during warmer periods is also supported by the comparison of the SDMs between LIG and the present (**Figures 3**, **4**). After the Last Glacial Maximum (LGM), *J. regia* survived and grew spontaneously in almost completely isolated stands in Asia, from Xinjiang province of Western China through Central Asia to the Caucasus (Pollegioni et al., 2017). ABC analyses have to be interpreted cautiously since the estimation of generation time for tree species is still controversial. The longevity of trees, the amount of generational overlap, the variable age of maturity, and the replacement speed of forests (Petit and Hampe, 2006) all vary over time and space (e.g., in cold–dry vs. warm–wet periods), complicating generation time estimates. In addition, the ABC method does not account for gene flow. Our results (and results of other studies) predicted strong gene flow between some subpopulations, which may result in under-estimates of generation time and the size of effective populations (Bai et al., 2014). Nevertheless, awareness of the possible effect of past climatic change on current populations may provide insight into this species’ future range dynamics in the light of climatic changes and be useful for germplasm management strategies (e.g., Lanier et al., 2015; Pollegioni et al., 2017; Ren et al., 2017).

### Multiple Refugia for Common Walnut in China

During LGM, *J. regia* was, with high probability (i.e., Prob. >0.75), distributed in Southern China, Northern China, and Central China (Qinling Mountains) (**Figure 3**). This result, as well as the projected habitat at the LGM (**Figure 3**), indicated that multiple potential allopatric refugia existed in these areas for this species (**Figure 3**). In this respect, results of the SDM were consistent with ABC analysis in terms of the general locations of refugia for common walnut. The current structure reflects expansion from ancient refugia (**Figures 1**, **3**, **4**). Based on fossil and genetic evidence, common walnut survived in multiple refugia in Europe (Pollegioni et al., 2014, 2017). Thus a model where common walnut expanded from multiple, disconnected refugia located in isolated suitable habitats is already accepted for other parts of the species range. Asian butternuts offer a strong case for the existence of a biogeographic divide between the northern and southern parts of East Asia during the Neogene and into the Pleistocene (Bai et al., 2014). A similar divide was observed for ecotypes of common walnut (*J. regia*, a northern and a southern refugium that were genetically distinct) (**Figure 1**; Han et al., 2016). The presence of the divide is also indicated by the large number of private alleles in Southern and Northern China (**Figure 2**). These private alleles may have been the result of bottlenecks followed by founder effects that resulted in the restriction of certain alleles to specific lineages. Other authors have argued that temperate forests retreated southward to c. 30°N during the Quaternary glacial periods as revealed by paleo-vegetation data from East Asia (Harrison et al., 2001), so the Northern Asian populations must have recolonized from southern glacial refugia. For example, distinct populations in Northern vs. Southern China that were not reflected in the spatial distribution of phenylalanine ammonia lyase (*PAL*) haplotypes (Han et al., 2016).

We conclude that although the number and location of refugia for temperate angiosperm trees in China is uncertain, there appear to have been multiple refugia containing *J. regia*. Evidence from our chloroplast studies, niche models, barrier analysis, and analysis of genetic structure at nuclear loci all point to multiple refugia for *J. regia* in China, some of which were likely shared by other *Juglans* species. The most important refugia for *J. regia* appear to have been located in Xinjiang province, in Northeast China (Bai et al., 2010, 2017), in the Qinling-Bashan Mountains (Bai et al., 2014), and Southwestern China (**Figures 1**, **4**, **5**). Han et al. (2016) suggested that Southwestern China, Qinling-Daba Mountains, and Northeastern China should be regarded as high priority areas for conservation of *J. regia* because of the rich genetic diversity found there. Our results indicated that Southwestern China and Northeastern China harbored the highest number of alleles, heterozygosity, and frequency of private allele (**Figure 2**).

### The Dispersal of *J. regia* in China

Theories about the introduction of *J. regia* into China have focused on two likely sources: Central Asia and parts of Southern China in or near the Himalayas. Common walnut, according to these theories, was transported by humans from these centers of origin into China. These models cite as evidence legends about Zhang Qian and the long history of trade along the Persian Royal Road and Silk Road. Although there is no doubt that walnut was traded along the silk road, a recent introduction of *J. regia* into China is not consistent with the genetic analysis (Aradhya et al., 2017; Pollegioni et al., 2017). It is likely that small-scale, long-distance dispersal of *J. regia* into China took place, probably from multiple sources: from Iran and Trans-Caucasus, Central Asia, and Western China (Pollegioni et al., 2015), and from wild populations in the Himalayas (Pollegioni et al., 2017). Sequencing of chloroplast haplotypes from a wider set of samples from Asia and China could shed light on this question. We believe, however, that the weight of evidence now favors the hypothesis that these introductions supplemented natural populations of *J. regia* that had a long and complex evolutionary history in China.

Evidence that *J. regia* is native to China includes the presence of chloroplasts that are predicted to have split from the Xinjiang and Tibetan *J. regia* as long ago as 2.7 Mya (**Figures 1**, **4**). We also conclude based on our niche models that habitat for *J. regia* was present in China continuously throughout the Pleistocene. Dode (1909) named several species, including *J. sinensis* (Northern China) and *J. duclouxiana* (Southern China) that are no longer recognized. Nevertheless, these former species, which were considered native plants by Dode, probably reflect indigenous *J. regia* populations. We identified Qinling Mountains (Central China) and Changbai Mountains (Northeast China) as the most likely locations for refugia for *J. regia* (and possibly other *Juglans* species native to China) based on their correspondence with refugia for related species (*J. cathayensis* and *J. mandshurica*; Bai et al., 2010, 2014; Wang et al., 2016) and the ENMs (**Figures 2**–**4**). The presence of high levels of genetic diversity in Chinese *J. regia* and strong regional genetic structure also favor an hypothesis that *J. regia* has a long evolutionary history in China. There is, in fact, little scientific evidence that indicates *J. regia* is not native to China. The presence of population A in Northeastern and Western China, and its geographic coincidence with ancient capital cities and roads (**Figure 4D**) may indicate human dispersal was responsible for the disjunction in the distribution of this genetic cluster. But the presence of suitable habitat for common walnut near Beijing during the previous LGM (**Figures 3**, **4**) increases uncertainty about how long common walnut has been present in Northeastern China (Bai et al., 2016, 2017).

It is not yet possible to untangle natural gene flow over evolutionary time and recent, human-mediated gene flow to determine what traits characterized common walnut in China 0.5 Mya or 0.1 Mya. Although humans can be efficient long-distance dispersers, it is not certain that their dispersal of common walnut into or across China fundamentally altered the genetic structure of the species formed during the Quaternary.

## Conclusion

We combined genetic data and SDMs to determine the genetic diversity and population structure of *J. regia* in China, estimate the degree of population differentiation among *J. regia* populations from different regions, and, finally, based on this data, describe the evolutionary history, most likely locations of glacial refugia, and the phytogeographic history of common walnut in China. Our analyses demonstrate that the present spatial genetic structure of walnut in China resulted from combined effects of expansion and contraction from multiple refugia after the LGM. We suggest that in addition to the well-attested ancient lineage of common walnut in Xinjiang province, there were additional refugia in Central, Southwest, and Northeast China throughout the Quaternary glaciations in China. Past climate changes affected intraspecific divergence of this species and led to genetic patterns that are important to understand the species’ current distribution and local adaptation. The evolutionary history of walnut is inextricably linked with human cultivation and dispersal. The relationship between humans and common walnut is exemplified by the impact of future climate changes and human management on population dynamics.

## Data Archiving

The whole chloroplast genome data deposited in the National Center for Biotechnology Information (NCBI) GenBank (Hu et al., 2016, 2017; accession numbers, KT963008, KX671976, KX671977, KX671975, and KT963008). The haplotypes of amino acid positions based on chloroplast genomes were submitted to the National Center for Biotechnology Information (NCBI). The accession numbers were MF953875–MF953885. The Unigene sequences and BLAST search results for 22 SSR-containing ESTs were provided as **Supplementary Tables S2**, **S5**.

## Author Contributions

PZ, XF, MM, and KW designed and performed the experiment as well as drafted the manuscript. XF, HZ, SZ, YH, LF, and PZ collected the samples. XF, HZ, LF, SZ, and PZ completed the sequence assembly and analyzed the data. XF, XL, LF, KW, and PZ conceived the study and revised the manuscript. All the authors have read and approved the final manuscript.

## Conflict of Interest Statement

The authors declare that the research was conducted in the absence of any commercial or financial relationships that could be construed as a potential conflict of interest.

## Footnotes

1 http://www.cvh.ac.cn/

2 http://www.nsii.org.cn/

3 http://www.worldclim.org/

## Supplementary Material

FIGURE S1 Detection of loci under selection from genome scans based on *F*_ST_ (Tsuda et al., 2015).

FIGURE S2 Genetic structure of 31 Chinese demes of *J. regia* based on 13 neutral SSR loci **(A)** locations of the 31 populations of *J. regia* and their color-coded grouping at the most likely *K* = 3. Purple, population A; Yellow, population B; Green, population C. **(B)** The proportion of the membership coefficient for each individual in the 31 *J. regia* demes for the inferred clusters when *K* = 3 according to STRUCTURE analysis. **(C)** The mean posterior probability value of structure results. **(D)** Principal coordinate analyses (*PCoA*) of 31 common walnut (*J. regia*) demes resolved into three genotype groups based on 14 neutral SSR loci. Colored circle based on clusters when *K* = 3 according to STRUCTURE analysis.

FIGURE S3 The 13 scenarios used for DIYABC analysis. The colors indicate population A, B, and C. red = purple, yellow = yellow, green = blue.

FIGURE S4 The PCA analysis based on the posterior distributions of DIYABC scenarios.

FIGURE S5 Principal coordinate analyses (*PCoA*) of 31 common walnut (*J. regia*) demes based on 22 microsatellite loci. Colored circles based on proportion of individuals assigned to each genetic clusters when *K* = 4according to STRUCTURE analysis (**Figure 5**).

FIGURE S6 Correlation between genetic and geographic distance based on IBD software (*r* = 0.5608, *P* < 0.001; Bohonak, 2002).

FIGURE S7 Genetic structure of 31 Chinese demes of *J. regia* based on 9 non-neutral SSR loci **(A)** locations of the 31 populations of *J. regia* and their color-coded grouping at the most likely *K* = 3. Purple, population I; Yellow, population II; Blue, population III. **(B)** The proportion of the membership coefficient for each individual in the 31 *J. regia* demes for the inferred clusters when *K* = 3 according to STRUCTURE analysis. **(C)** The mean posterior probability value of structure results.

FIGURE S8 Estimated contemporary gene flow among three populations (A, B, and C) using software BAYESASS (Wilson and Rannala, 2003).

TABLE S1 Population’s sites, sample size(s) and values of the genetic diversity parameters based on 31 *J. regia* populations.

TABLE S2 EST-SSRs used in this study, their gene context and polymorphism.

TABLE S3 Nine non-neutral EST-SSR loci, their gene context and polymorphism.

TABLE S4 *P*-value for genotypic disequilibrium based on 156,860 permutations.

TABLE S5 Prior distributions of the parameters used in DIYABC.

TABLE S6 Demographic parameters of scenario 3 obtained by DIYABC.

TABLE S7 Summary of 19 bioclimatic layers used in niche models.

TABLE S8 Summary of Chi-Square tests for Hardy–Weinberg equilibrium.

TABLE S9 Summary of private alleles by population.

TABLE S10 List of samples with one or more private alleles.

TABLE S11 Pairwise *F*_ST_ values between 31 populations.
