# Supplementary figures and images for: The Phytogeographic History of Common Walnut in China
Source: Front Plant Sci. 2018 Sep 21;9:1399. doi: 10.3389/fpls.2018.01399 (PMC6160591; doi:10.3389/fpls.2018.01399)

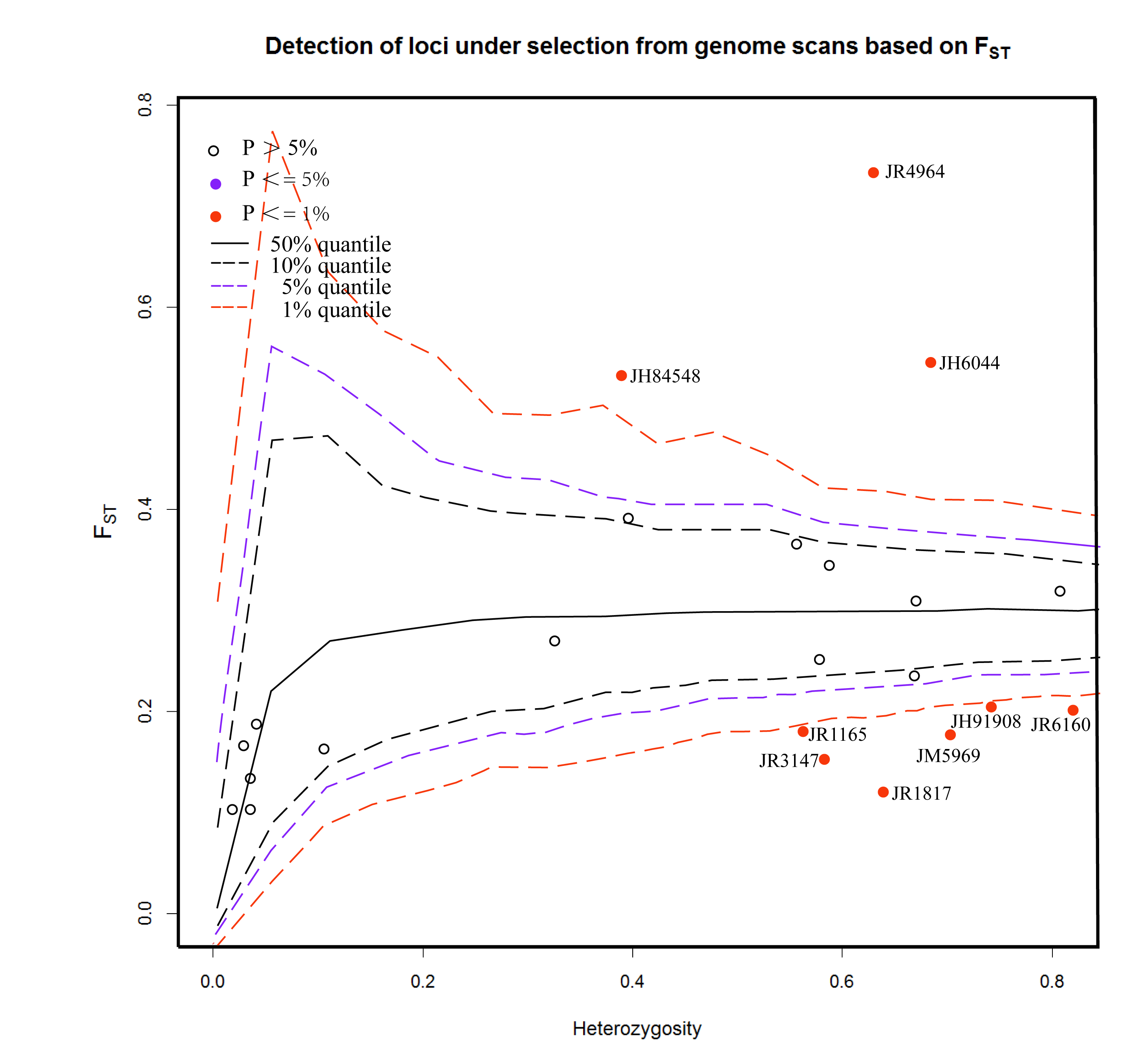

Supplement: FIGURE S1 — Detection of loci under selection from genome scans based on FST (Tsuda et al., 2015). [file Image_1.TIF]

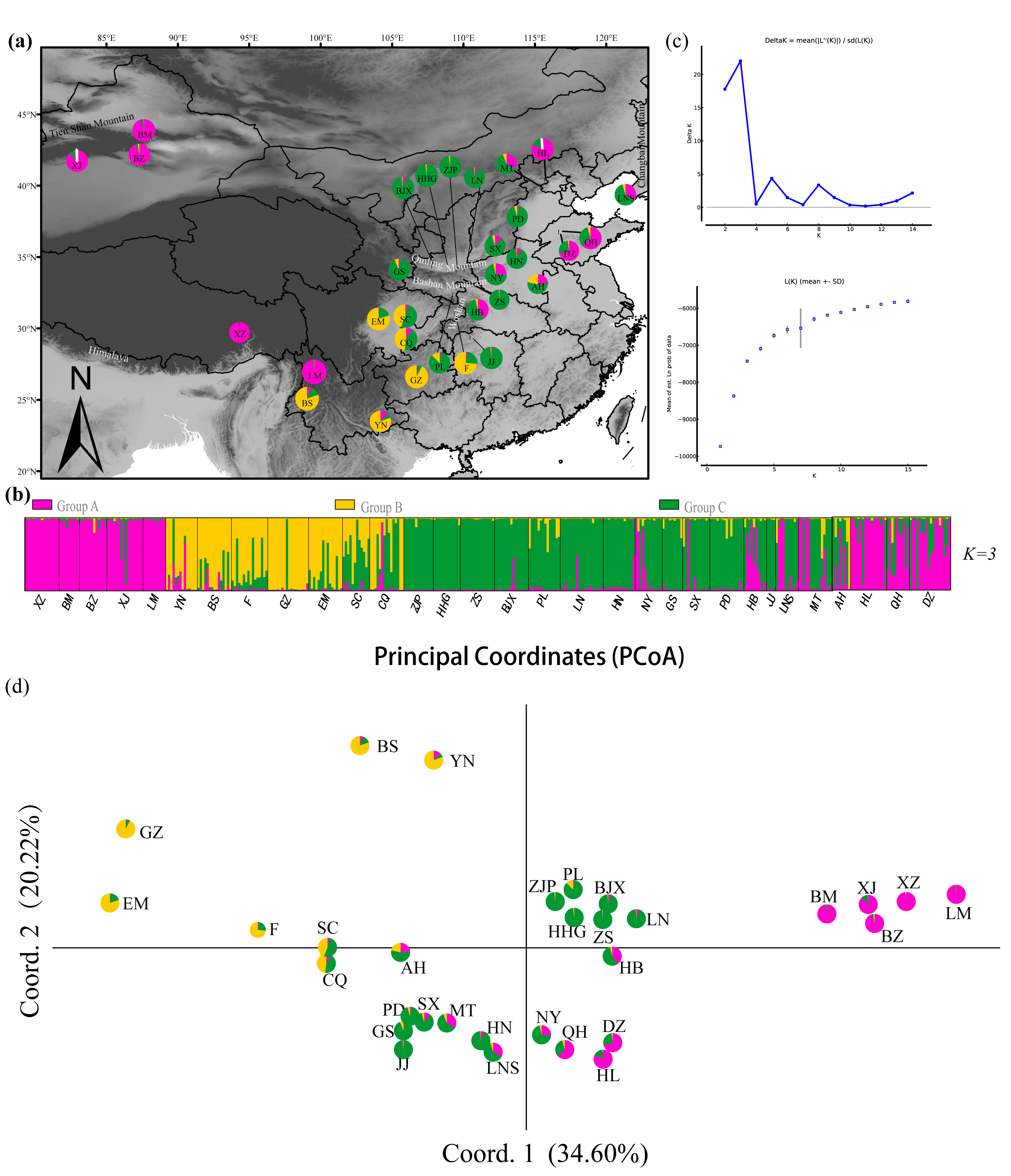

Supplement: FIGURE S2 — Genetic structure of 31 Chinese demes of J. regia based on 13 neutral SSR loci (A) locations of the 31 populations of J. regia and their color-coded grouping at the most likely K = 3. Purple, population A; Yellow, population B; Green, population C. (B) The proportion of the membership coefficient for each individual in the 31 J. regia demes for the inferred clusters when K = 3 according to STRUCTURE analysis. (C) The mean posterior probability value of structure results. (D) Principal coordinate analyses (PCoA) of 31 common walnut (J. regia) demes resolved into three genotype groups based on 14 neutral SSR loci. Colored circle based on clusters when K = 3 according to STRUCTURE analysis. [file Image_2.TIF]

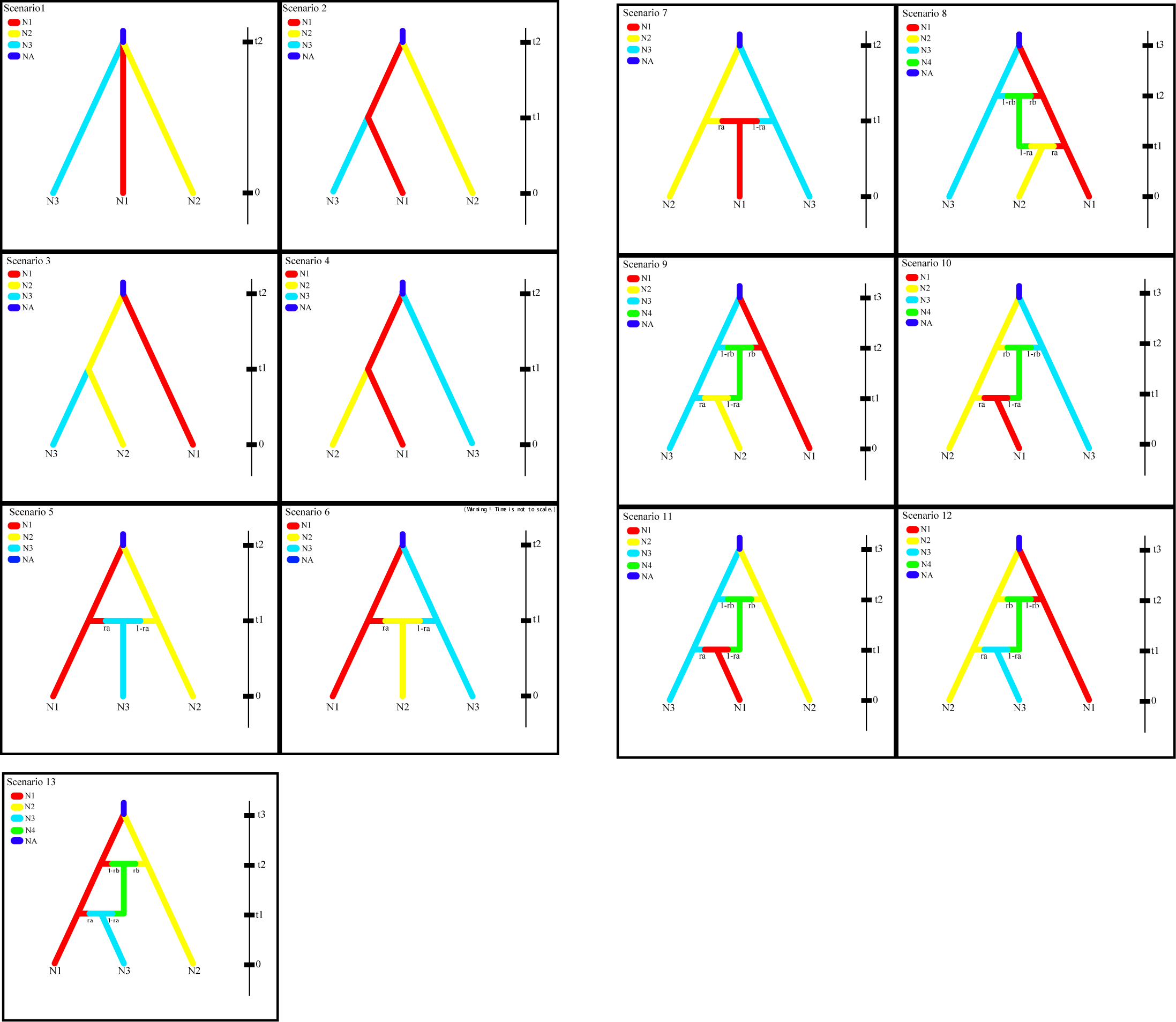

Supplement: FIGURE S3 — The 13 scenarios used for DIYABC analysis. The colors indicate population A, B, and C. red = purple, yellow = yellow, green = blue. [file Image_3.TIF]

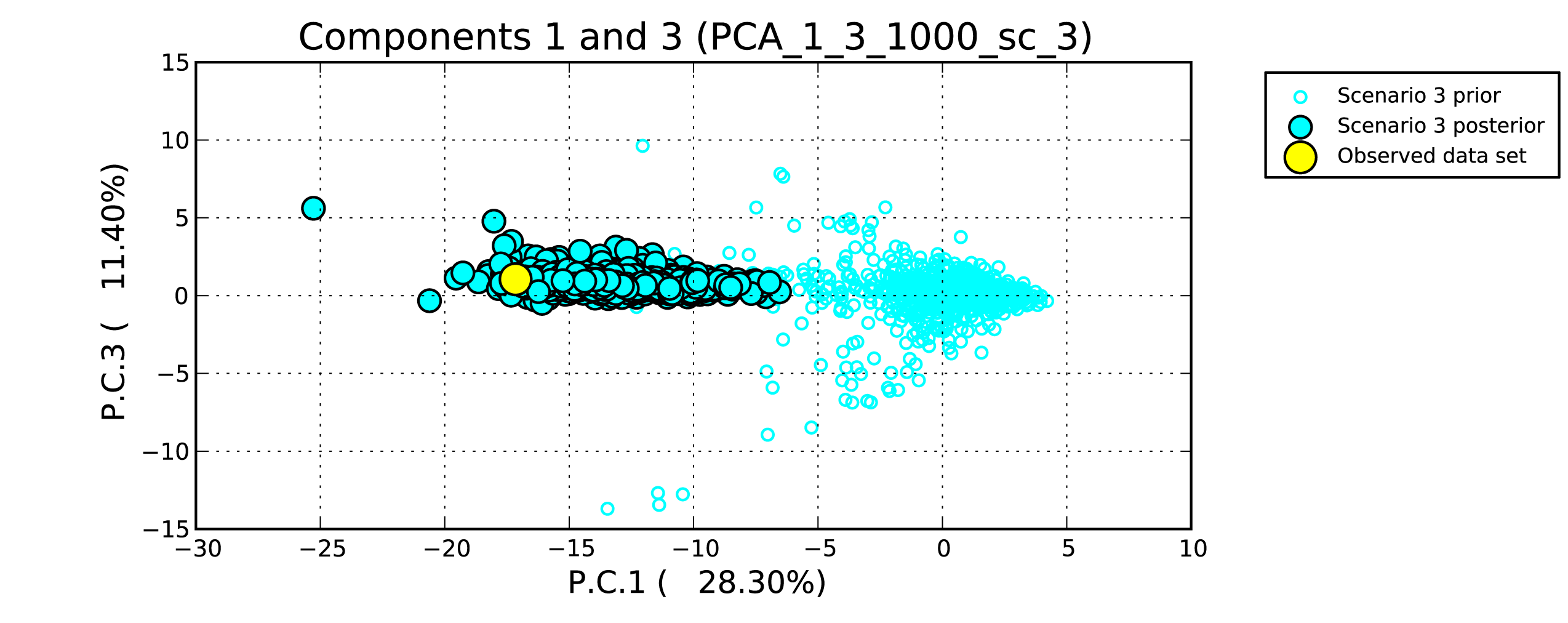

Supplement: FIGURE S4 — The PCA analysis based on the posterior distributions of DIYABC scenarios. [file Image_4.TIF]

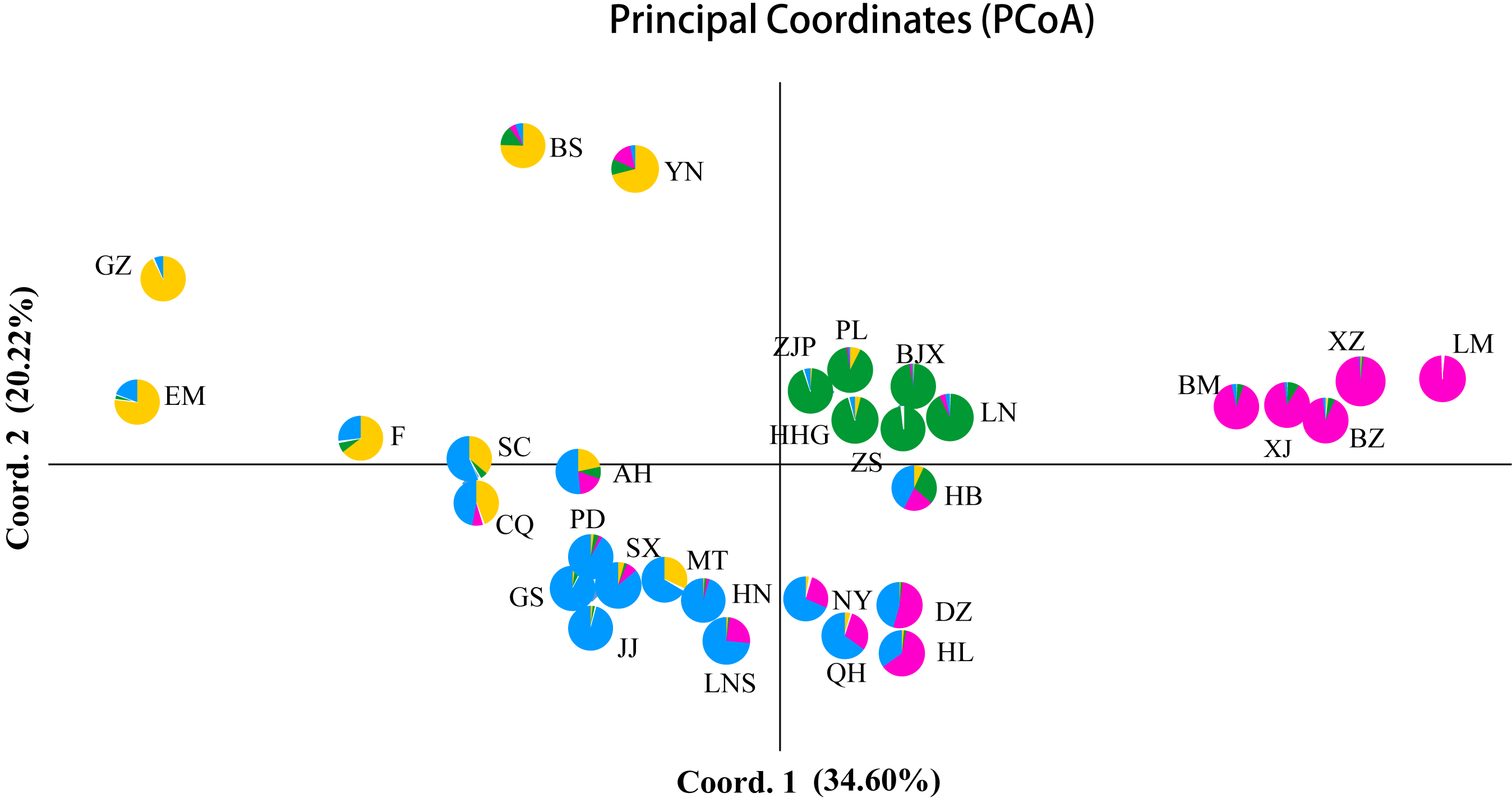

Supplement: FIGURE S5 — Principal coordinate analyses (PCoA) of 31 common walnut (J. regia) demes based on 22 microsatellite loci. Colored circles based on proportion of individuals assigned to each genetic clusters when K = 4according to STRUCTURE analysis (Figure 5). [file Image_5.TIF]

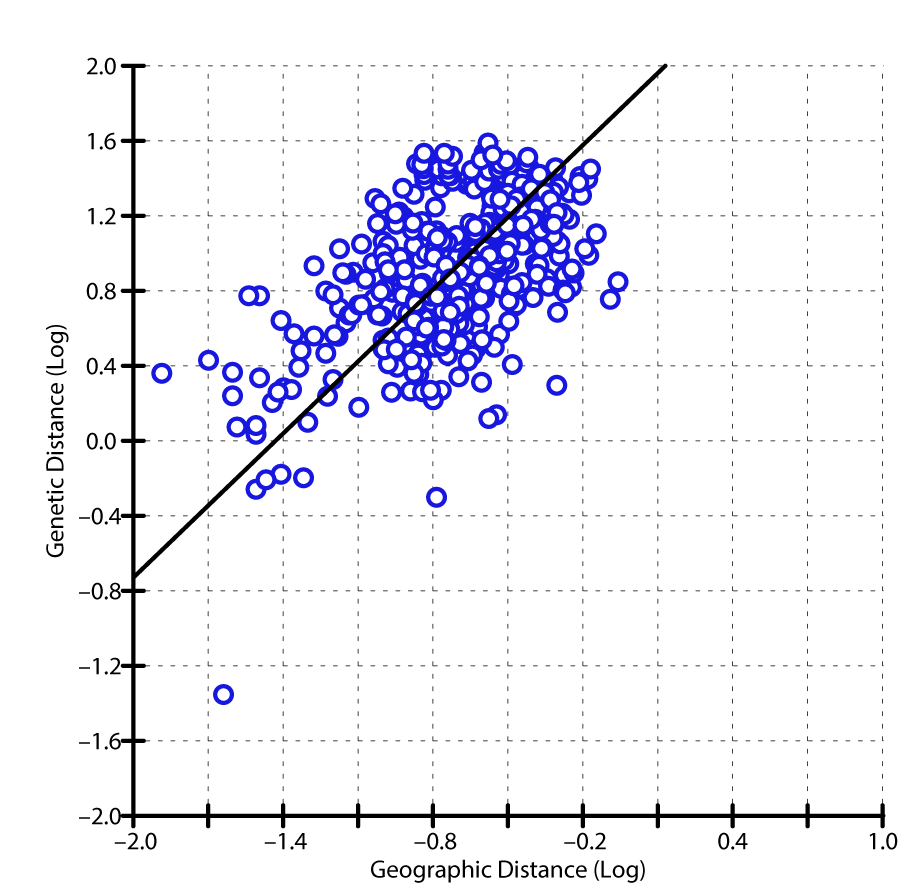

Supplement: FIGURE S6 — Correlation between genetic and geographic distance based on IBD software (r = 0.5608, P < 0.001; Bohonak, 2002). [file Image_6.TIF]

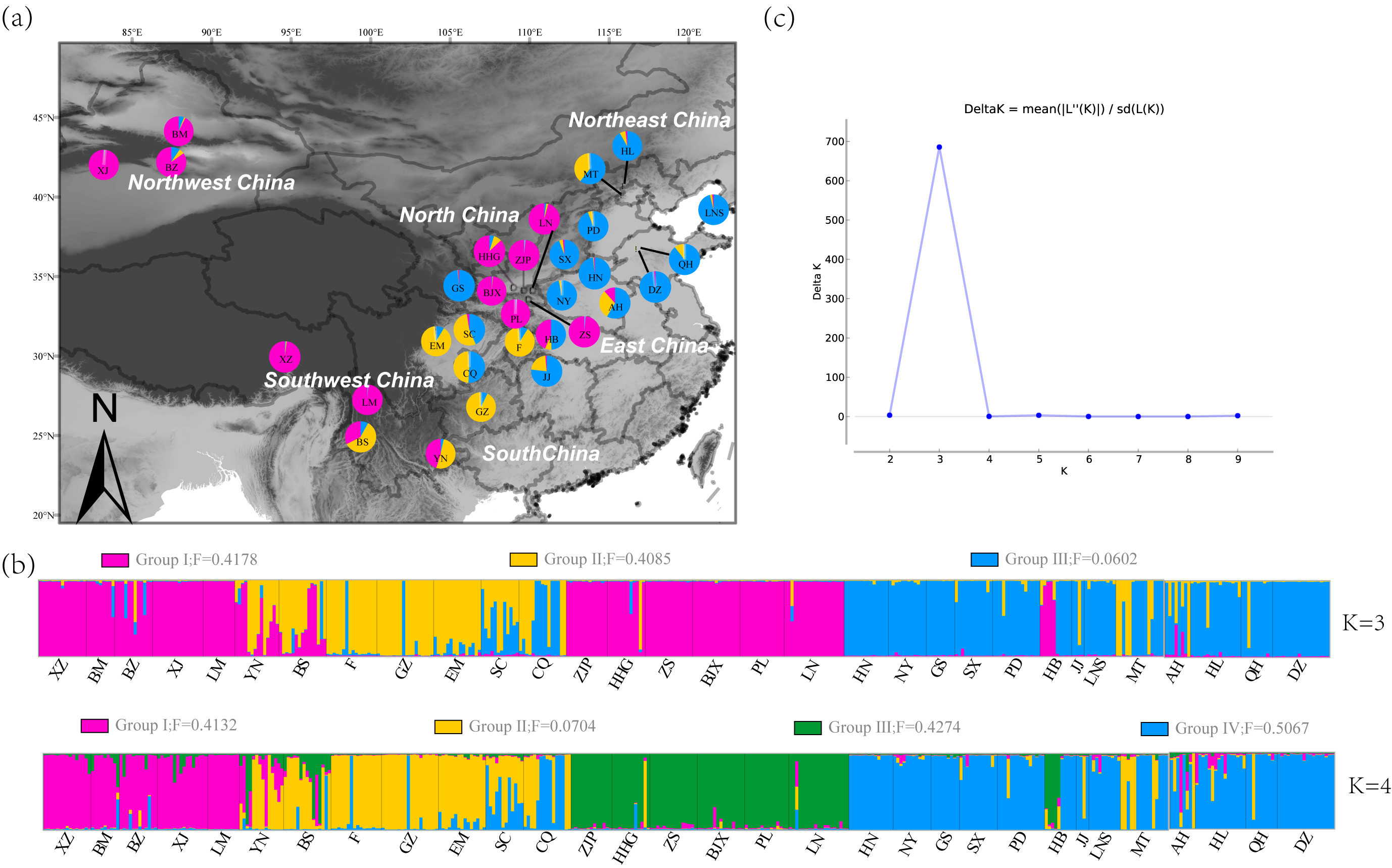

Supplement: FIGURE S7 — Genetic structure of 31 Chinese demes of J. regia based on 9 non-neutral SSR loci (A) locations of the 31 populations of J. regia and their color-coded grouping at the most likely K = 3. Purple, population I; Yellow, population II; Blue, population III. (B) The proportion of the membership coefficient for each individual in the 31 J. regia demes for the inferred clusters when K = 3 according to STRUCTURE analysis. (C) The mean posterior probability value of structure results. [file Image_7.TIF]

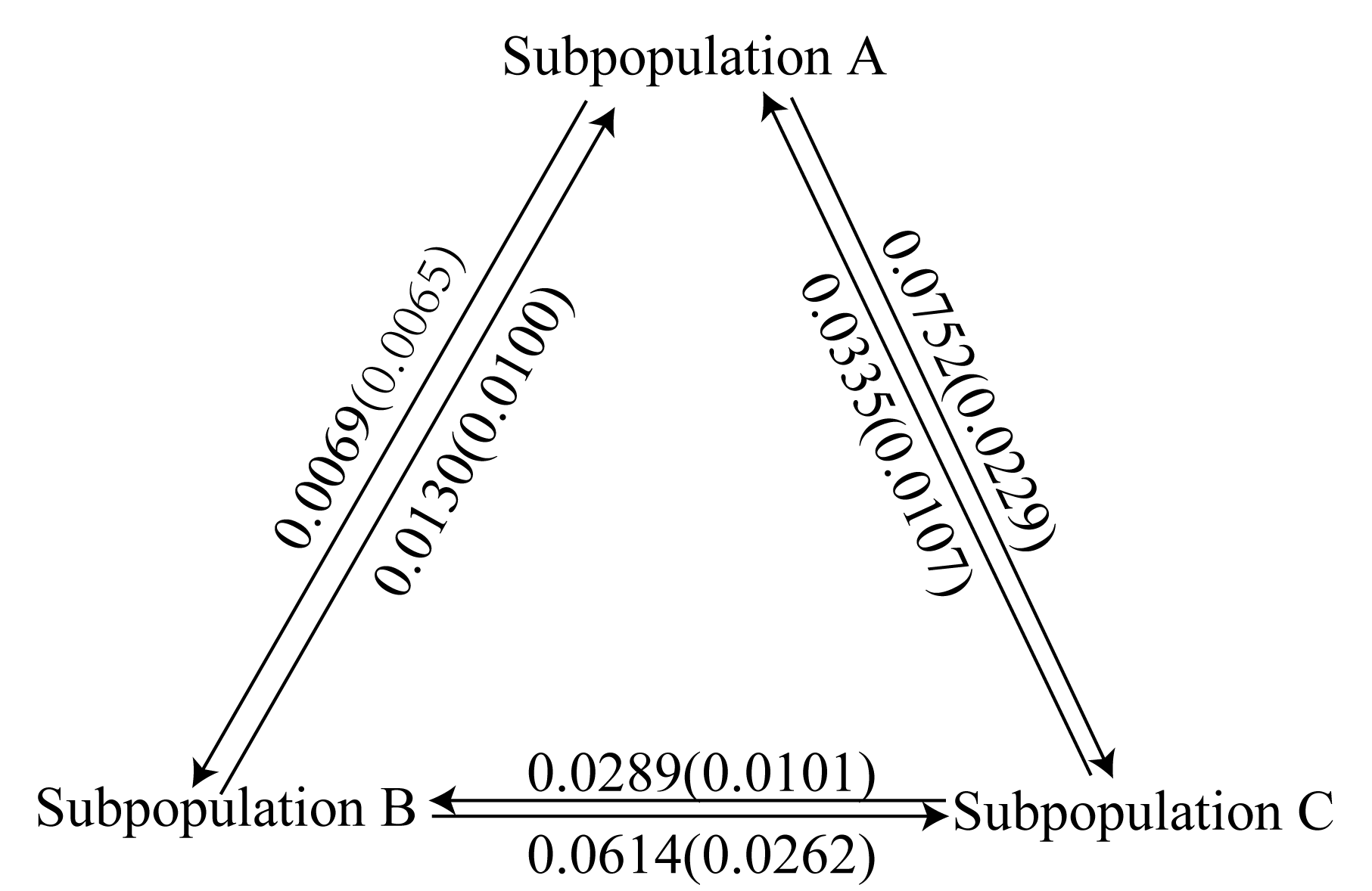

Supplement: FIGURE S8 — Estimated contemporary gene flow among three populations (A, B, and C) using software BAYESASS (Wilson and Rannala, 2003). [file Image_8.TIF]
