# Supplementary material for: The Phytogeographic History of Common Walnut in China
Source: Front Plant Sci. 2018 Sep 21;9:1399. doi: 10.3389/fpls.2018.01399 (PMC6160591; doi:10.3389/fpls.2018.01399)
Supplement: TABLE S1 — Population’s sites, sample size(s) and values of the genetic diversity parameters based on 31 J. regia populations. [file Table_1.DOC]

**Table S1.** Population’s sites, sample size(S) and values of the genetic diversity parameters based on 31 *J. regia* populations

| Site | Pop | Samples | longitude(E) | latitude(N) | Na | Ne | Rs | I | *PPL* (%) | H_O_ | H_E_ | *F*_IS_ |
| --- | --- | --- | --- | --- | --- | --- | --- | --- | --- | --- | --- | --- |
| Heshuo, Xinjiang | BM^A/I^ | 14^a^/1^b^ | 87.63 | 43.79 | 2.17 | 1.74 | 1.33 | 0.52 | 65.22 | 0.24 | 0.31 | 0.23 |
| Heshuo, Xinjiang | BZ^A/I^ | 18/0 | 87.31 | 42.23 | 2.17 | 1.65 | 1.29 | 0.47 | 60.87 | 0.21 | 0.27 | 0.23 |
| Qihe, Shandong | DZ^A/I^ | 27/3 | 116.77 | 36.74 | 2.65 | 1.77 | 1.30 | 0.53 | 60.87 | 0.22 | 0.29 | 0.26 |
| Zhangjiakou, Hebei | HL^A/I^ | 23/0 | 115.8 | 40.34 | 2.74 | 1.96 | 1.33 | 0.58 | 60.87 | 0.24 | 0.31 | 0.19 |
| Lijiang, Yunnan | LM^A/I^ | 15/0 | 99.68 | 27.03 | 1.26 | 1.14 | 1.08 | 0.12 | 26.09 | 0.13 | 0.08 | -0.45 |
| Qihe, Shandong | QH^A/I^ | 15/0 | 116.76 | 36.78 | 2.61 | 1.88 | 1.31 | 0.55 | 65.22 | 0.19 | 0.30 | 0.36 |
| Kuche, Xinjiang | XJ^A/I^ | 24/1 | 82.96 | 41.72 | 2.22 | 1.55 | 1.27 | 0.44 | 69.57 | 0.22 | 0.26 | 0.15 |
| Linzhi, Tibet | XZ^A/I^ | 23/1 | 94.36 | 29.65 | 2.17 | 1.53 | 1.29 | 0.46 | 65.22 | 0.22 | 0.28 | 0.22 |
| Baoshan, Yunnan | BS^B/II^ | 23/1 | 99.17 | 25.12 | 2.22 | 1.7 | 1.30 | 0.5 | 65.22 | 0.27 | 0.29 | 0.18 |
| Emei, Sichuan | EM^B/II^ | 23/0 | 104.08 | 30.65 | 2.87 | 1.96 | 1.38 | 0.66 | 73.91 | 0.28 | 0.37 | 0.2 |
| Fengjie, Chongqing | F^B/II^ | 23/1 | 109.46 | 31.02 | 2.39 | 1.67 | 1.32 | 0.53 | 69.57 | 0.4 | 0.31 | -0.19 |
| Guizhou | GZ^B/II^ | 27/2 | 106.71 | 26.6 | 4.09 | 2.19 | 1.41 | 0.8 | 82.61 | 0.26 | 0.40 | 0.35 |
| Nanchong,Sichuan | SC^B/II^ | 18/0 | 105.91 | 30.87 | 2.96 | 1.84 | 1.36 | 0.64 | 73.91 | 0.29 | 0.34 | 0.12 |
| Wenshan, Yunnan | YN^B/II^ | 20/0 | 104.22 | 23.40 | 3.09 | 1.98 | 1.42 | 0.72 | 73.91 | 0.25 | 0.40 | 0.39 |
| Fuyang, Anhui | AH^C/III^ | 15/0 | 115.26 | 33.04 | 2.17 | 1.8 | 1.38 | 0.55 | 65.22 | 0.16 | 0.33 | 0.45 |
| Yongchuan, Chongqing | CQ^C/III^ | 23/0 | 105.93 | 29.36 | 3.48 | 2.19 | 1.43 | 0.78 | 78.26 | 0.25 | 0.42 | 0.42 |
| Tianshui, Gansu | GS^C/III^ | 14/1 | 106.05 | 34.3 | 1.91 | 1.5 | 1.25 | 0.4 | 56.52 | 0.19 | 0.24 | 0.15 |
| Zhenzhou, Henan | HN^C/III^ | 21/1 | 113.75 | 34.77 | 2.09 | 1.51 | 1.26 | 0.42 | 60.87 | 0.23 | 0.25 | 0.06 |
| Zhangjiajie, Hunan | JJ^C/III^ | 6/1 | 110.69 | 29.37 | 1.52 | 1.33 | 1.20 | 0.26 | 39.13 | 0.23 | 0.17 | -0.36 |
| Dalian, Liaoning | LNS^C/III^ | 11/0 | 121.62 | 38.89 | 2.09 | 1.63 | 1.32 | 0.49 | 69.57 | 0.17 | 0.30 | 0.42 |
| Mentougou, Beijing | MT^C/III^ | 23/0 | 115.48 | 39.96 | 3.43 | 2.25 | 1.51 | 0.89 | 95.65 | 0.21 | 0.50 | 0.59 |
| Nanyang, Henan | NY^C/III^ | 15/0 | 111.74 | 33.62 | 2.22 | 1.73 | 1.33 | 0.52 | 69.57 | 0.22 | 0.31 | 0.22 |
| Pingding, Shanxi | PD^C/III^ | 21/0 | 113.88 | 37.78 | 2.26 | 1.69 | 1.28 | 0.47 | 56.52 | 0.18 | 0.27 | 0.26 |
| Qinshui, Shanxi | SX^C/III^ | 18/1 | 112.19 | 35.69 | 2.17 | 1.53 | 1.28 | 0.45 | 69.57 | 0.2 | 0.27 | 0.27 |
| Shangluo, Shaanxi | ZS^C/III^ | 23/0 | 109.88 | 33.53 | 2.09 | 1.61 | 1.29 | 0.48 | 56.52 | 0.24 | 0.28 | 0.14 |
| Baoji, Shaanxi | BJX^C/IV^ | 23/1 | 107.87 | 34.36 | 2.22 | 1.56 | 1.28 | 0.45 | 60.87 | 0.21 | 0.27 | 0.2 |
| Yichang, Hubei | HB^C/IV^ | 15/1 | 110.76 | 31.23 | 3.13 | 1.98 | 1.39 | 0.69 | 82.61 | 0.21 | 0.37 | 0.38 |
| Honghegu, Shaanxi | HHG^C/IV^ | 18/0 | 108.95 | 34.27 | 2.26 | 1.52 | 1.28 | 0.45 | 65.22 | 0.2 | 0.27 | 0.25 |
| Luonan, Shaanxi | LN^C/IV^ | 29/0 | 110.15 | 34.09 | 2.13 | 1.6 | 1.30 | 0.47 | 69.57 | 0.23 | 0.29 | 0.19 |
| Pingli, Shaanxi | PL^C/IV^ | 21/1 | 109.36 | 32.39 | 2.39 | 1.68 | 1.29 | 0.49 | 60.87 | 0.27 | 0.28 | 0.02 |
| Lantian, Shaanxi | ZJP^C/IV^ | 20/0 | 109.6 | 34.1 | 2.35 | 1.64 | 1.30 | 0.49 | 69.57 | 0.25 | 0.29 | 0.16 |
| Average |  |  |  |  | 2.43 | 1.72 |  | 0.53 | 65.78 | 0.23 | 0.30 | 0.23 |

^a^Indicates the number of samples used for analysis using SSRs

^b^Indicates the number of samples used for whole chloroplast genome sequencing.

Population cod (pop), "A", " B", and "C" indicated metapopulations of ABC analysis as identified using STRUCTURE analysis based on 13 neutral SSR loci (Fig. S2). Population code (pop), "I", "II", "III", and "IV" indicate metapopulations as identified using STRUCTURE analysis based on 22 SSR loci (13 neutral and 9 non-neutral ) (Fig. 5), number of samples (S), the number of alleles (Na), number of effective alleles (Ne), allelic richness (*R_S_*) Shannon's Information Index (I), percentage of polymorphic loci (PPL), observed Heterozygosity (H_O_), expected heterozygosity (H_E_) and fixation index (*F*_IS_) in 31populations.
