# Supplementary material for: The Phytogeographic History of Common Walnut in China
Source: Front Plant Sci. 2018 Sep 21;9:1399. doi: 10.3389/fpls.2018.01399 (PMC6160591; doi:10.3389/fpls.2018.01399)
Supplement: TABLE S2 — EST-SSRs used in this study, their gene context and polymorphism. [file Table_2.DOC]

**Table S2.** EST-SSRs used in this study, their gene context and polymorphism.

| Locus | Sequence (5'-3') | Repeat | *Na* | Putative function of Unigene | Reference |
| --- | --- | --- | --- | --- | --- |
| JC8125 | F: AGCAACCAGAGCAGAGCATT | (TCT)_7_ | 11 | Transcription factor AtMYC2, putative [*Ricinuscommunis*] (query length, 2642 bp; query cover,77 %; E value,0; ident, 66 %) | Dang et al. 2015 |
|  | R: AACCTCAACACCAACTATGCT |  |  |  |  |
| JH89978 | F:ACCTTCCCTGCTCCTCTCTT | (GGT)_6_ | 5 | Zinc ion binding, putative isoform 1 [*Theobroma cacao*] (query length, 4410 bp; query cover,84 %; E value,0; ident, 62 %) | Hu et al. 2015 |
|  | R: GAGCCTTGTGGAAGCAAACG |  |  |  |  |
|  |  |  |  |  |  |
| JC7329 | F: TGCAGCGCATCAGTGAGTTA | (TGA)_8_ | 2 | Hypothetical protein CISIN_1g001847mg [*Citrus sinensis*] (query length, 4233 bp; query cover,62 %; E value,0; ident, 79 %) | Dang et al. 2015 |
|  | R: ACGCTCGAGTGTAGTAGCAAG |  |  |  |  |
| JM61666 | F: AACTGTTGCCGGAGCTTTCT | (GA)_11_ | 3 | Salt tolerance-like family protein [*Populustrichocarpa*] (query length, 1435 bp; query cover,49 %; E value,5e-132; ident, 82 %) | Hu et al. 2015 |
|  | R: TGGGATAACACCACATGCAGT |  |  |  |  |
| JR4616 | F:AGCCCTTTTGCATCGGCTAT | (AGAC)_5_ | 6 | PREDICTED: zinc finger CCCH domain-containing protein 18-like isoform X3 [*Citrus sinensis*] (query length, 2396 bp; query cover,72 %; E value,0; ident, 73 %) | Dang et al. 2016 |
|  | R:AGCTGACCGATCGATCAACA |  |  |  |  |
|  |  |  |  |  |  |
| JC5411 | F:AAGCTGTTTGTGCCAAAAGC | (GAT)_7_ | 3 | Fructose 1,6 bisphosphate aldolase class 1 [*Carica papaya*] (query length, 1624 bp; query cover,73 %; E value,0; ident, 93 %) | Dang et al. 2015 |
|  | R:TTCTAGCGAGAATTCCGGCC |  |  |  |  |
| JC2995 | F: AACTGTTGCCGGAGCTTTCT | (GA)_10_ | 4 | COL domain class transcription factor isoform 1 [*Theobroma cacao*] (query length, 1510 bp; query cover,47 %; E value,3e-133; ident, 82 %) | Dang et al. 2015 |
|  | R: TGGGATAACACCACATGCAGT |  |  |  |  |
| JH42753 | F:CAGTTTTGGCCAGCTGCAAT | (GCT)_6_ | 10 | PREDICTED: uncharacterized protein LOC102709351 [*Oryzabrachyantha*] (query length, 480 bp; query cover,23 %; E value,4e-13; ident, 92 %) | Hu et al. 2015 |
|  | R: TGTGCCCATGCTAAGACTGG |  |  |  |  |
| JH86514 | F:CGTTACGTCGGGAGGATGAG | (TTAGGG)_6_ | 8 | Ubiquitin-conjugating enzyme/RWD-like protein [*Theobroma cacao*] (query length, 1479 bp; query cover,79 %; E value,3e-171; ident, 70 %) | Hu et al. 2015 |
|  | R: CCTCGTTCGTAGTCTCAGCC |  |  |  |  |
| JR3773 | F:GGTGGTTTGACCCTTAATTCTGT | (CTGT)_5_ | 5 | None (query length,506 bp) | Dang et al. 2016 |
|  | R:ACCCTGCCACAATGACCAAA |  |  |  |  |
| JM78331 | F: GCAGTGCGCTCCTTTTTCAA | (AACGGC)_5_ | 4 | Thymidylate synthase 1 isoform 6 [*Theobroma cacao*] (query length, 2593 bp; query cover,53 %; E value,2e-160; ident, 82 %) | Hu et al. 2015 |
|  | R: TTCTCGGGTTGAAGCCACAA |  |  |  |  |
| JM68820^a^ | F: TCCTTCTGTGTGAGTGCGTG | (ACAT)_14_ | 15 | Hypothetical protein CICLE_v10032529mg [*Citrus clementina*] (query length, 1726 bp; query cover,34 %; E value,2e-60; ident, 70 %) | Hu et al. 2015 |
|  | R: GGTCAGGTGAGTGGAGCAAA |  |  |  |  |
| JR6439 | F:TCGATGCGATCATCTCCGTG | (TGCG)_5_ | 11 | G2484-1 protein, putative isoform 5 [*Theobroma cacao*] (query length, 7506 bp; query cover,87 %; E value,0 ; ident, 60 %) | Dang et al. 2016 |
|  | R:CGGCACCAAAACAGAACTCG |  |  |  |  |
| JH2096 | F:AAGCTATGTTGGCTGCTGGT | (GCA)_7_ | 8 | Hypothetical protein PHAVU_008G242400g [*Phaseolus vulgaris*] (query length, 2260 bp; query cover,80 %; E value,0 ; ident, 79 %) | Hu et al. 2015 |
|  | R:ATTGTTCAGCGGTTGCCCTA |  |  |  |  |
| Average |  |  | 6.79 |  |  |

^a^Indicatesa locus that had highest probability null alleles than other loci, so it was deleted from all subsequent analyses (*P*-value<0.01).
