# Supplementary material for: The Phytogeographic History of Common Walnut in China
Source: Front Plant Sci. 2018 Sep 21;9:1399. doi: 10.3389/fpls.2018.01399 (PMC6160591; doi:10.3389/fpls.2018.01399)
Supplement: TABLE S3 — Nine non-neutral EST-SSR loci, their gene context and polymorphism. [file Table_3.DOC]

**Table S3.** Nine non-neutral loci used for analysis of genetics of common walnut in China, their gene context and polymorphism.

| Locus | Sequence (5'-3') | Repeat | *Na* | Putative function of Unigene | Reference |
| --- | --- | --- | --- | --- | --- |
| JM5969 | F: ACAATAGTCTCTGCACCGCC  R: AGCTTGTACTTACCGCCGAC | (AG)_10_ | 11 | Earlier flowering 1 [*Dimocarpus longan*] (query length, 2739 bp; query cover,45 %; E value,0; ident, 83 %) | Hu *et al*., 2015 |
| JR4964 | F:CTCGATCTGAACTCGGCTCC  R: TCTACTCTCTCCGCACCACA | (GGGA)_5_ | 7 | B-block TFIIIC Transcribes tRNA on eukaryotic RNA polymerase III | Dang *et al*., 2016 |
| JH91908 | F:GAAAAGCATGGTCCTGCTGC  R:ATTGAGCGACGAAAAGGGGT | (CTG)_7_ | 19 | PREDICTED: *Juglans regia* NAC domain-containing protein 62-like (LOC109007515), mRNA (query length, 689 bp; query cover,66 %; E value,0 ; ident, 98 %) | Hu *et al*., 2015 |
|  |  |  |  |  |  |
| JR1165 | F:CACGTAGCGTCCGTAATCGA  R:CAGCACCTCCACTAACTGCA | (AGAT)_6_ | 8 | *Juglans regia* nonspecific lipid transfer protein mRNA, complete cds (query length, 808 bp; query cover,44 %; E value,2e-155 ; ident, 95 %) | Dang *et al*., 2016 |
| JH84548 | F:TCTGAGGAAGCTGCATGGAA  R: AACTCTGGACACATGCCGC | (TGCA)_6_ | 5 | Histidine phosphotransfer (HPt) proteins are components of the histidine kinase (HK) to response regulator (RR) signal transduction scheme | Hu *et al*., 2015 |
| JR6160 | F:ACTTCAGGTTCCCAACGCAA  R:TAGAGGGAAGGTCTCCGGTG | (GA)_10_ | 16 | Mitogen-activated protein kinase (MAPK) family members connect cell surface receptors to regulatory targets within cells and influence a number of tissue-specific biological activities such as cell proliferation, differentiation and survival. | Dang *et al*., 2016 |
| JH6044 | F:CCTCGTCTCCTCCCCTAACA  R:GTAGGATAGTGTGGCGTCGG | (CCA)_7_ | 11 | Hypothetical protein CICLE_v10012640mg [*Citrus clementina*] (query length, 1096 bp; query cover,44 %; E value,3e-95 ; ident, 87 %) | Hu *et al*., 2015 |
| JR3147 | F:CAGCACCTCCACTAACTGCA  R:CACGTAGCGTCCGTAATCGA | (CTAT)6 | 11 | *Juglans regia* nonspecific lipid transfer protein mRNA, complete cds (query length, 860 bp; query cover,41 %; E value,9e-154 ; ident, 94 %) | Dang *et al*., 2016 |
| JR1817 | F: CCTCAGAGCCAACCATCCTT  R: AGAACAGAACCAGCGTCACA | (AC)_11_ | 8 | CO and FT orthologs, belonging to the BBX and PEBP family, respectively, photoperiod regulation of flowering time | Dang *et al*. 2016 |
