# Supplementary material for: The Phytogeographic History of Common Walnut in China
Source: Front Plant Sci. 2018 Sep 21;9:1399. doi: 10.3389/fpls.2018.01399 (PMC6160591; doi:10.3389/fpls.2018.01399)
Supplement: TABLE S5 — Prior distributions of the parameters used in DIYABC. [file Table_5.DOC]

**Table S5.** Prior distributions of the parameters used in DIYABC.

| Parametermean | mean | median | mode | q(2.5%） | q(5%) | q(25%) | q(75%） | q(95%) | q(97.5%) |
| --- | --- | --- | --- | --- | --- | --- | --- | --- | --- |
| N1 | 3.53E+05 | 3.02E+05 | 2.46E+05 | 6.67E+04 | 9.02E+04 | 1.93E+05 | 4.65E+05 | 7.98E+05 | 8.81E+05 |
| N2 | 4.09E+05 | 3.69E+05 | 3.45E+05 | 9.61E+04 | 1.24E+05 | 2.46E+05 | 5.38E+05 | 8.28E+05 | 8.96E+05 |
| N3 | 5.25E+05 | 5.10E+05 | 4.88E+05 | 1.42E+05 | 1.82E+05 | 3.56E+05 | 6.85E+05 | 9.11E+05 | 9.52E+05 |
| t1 | 2.30E+04 | 2.25E+04 | 2.59E+04 | 2.84E+03 | 4.25E+03 | 1.36E+04 | 3.17E+04 | 4.40E+04 | 4.65E+04 |
| t2 | 5.62E+04 | 5.58E+04 | 4.95E+04 | 1.35E+04 | 1.79E+04 | 3.75E+04 | 7.49E+04 | 9.48E+04 | 9.73E+04 |
| NA | 1.10E+05 | 5.66E+04 | 5.04E+03 | 2.58E+03 | 4.82E+03 | 2.38E+04 | 1.30E+05 | 4.19E+05 | 5.72E+05 |
| µmic_1 | 2.93E-06 | 2.14E-06 | 1.52E-06 | 1.08E-06 | 1.15E-06 | 1.56E-06 | 3.23E-06 | 6.98E-06 | 9.48E-06 |
| pmic_1 | 6.00E-01 | 6.26E-01 | 9.00E-01 | 1.73E-01 | 2.19E-01 | 4.47E-01 | 7.76E-01 | 8.96E-01 | 9.00E-01 |
| snimic_1 | 2.29E-07 | 4.17E-08 | 1.00E-08 | 1.01E-08 | 1.06E-08 | 1.81E-08 | 1.27E-07 | 9.14E-07 | 1.83E-06 |
