# Supplementary material for: The Phytogeographic History of Common Walnut in China
Source: Front Plant Sci. 2018 Sep 21;9:1399. doi: 10.3389/fpls.2018.01399 (PMC6160591; doi:10.3389/fpls.2018.01399)
Supplement: TABLE S6 — Demographic parameters of scenario 3 obtained by DIYABC. [file Table_6.DOC]

**Table S6.** Demographic parameters of scenario 3 obtained by DIYABC.

| Summary statistics | Observed value | Scenario3 |
| --- | --- | --- |
| Mean number of alleles in pop1 | 4.8667 | 0.0020 (**) |
| Mean number of alleles in pop2 | 5.6 | 0.0031 (**) |
| Mean number of alleles in pop3 | 6.7333 | 0.0033 (**) |
| Mean expected heterozygosity in pop1 | 0.3759 | 0.0012 (**) |
| Mean expected heterozygosity in pop2 | 0.3813 | 0.0013 (**) |
| Mean expected heterozygosity in pop3 | 0.3534 | 0.0010 (**) |
| *F*_ST_ (Pop1 and Pop2) | 0.1814 | 0.9919 (**) |
| *F*_ST_ (Pop1 and Pop3) | 0.1545 | 0.9893 (*) |
| *F*_ST_ (Pop2 and Pop3) | 0.0655 | 0.9742 (*) |
| Mean index of genotype likelihood(Pop1 and Pop2) | 1.1223 | 0.0005 (***) |
| Mean index of genotype likelihood(Pop1 and Pop3) | 1.136 | 0.0005 (***) |
| Mean index of genotype likelihood(Pop2 and Pop1) | 0.9529 | 0.0003 (***) |
| Mean index of genotype likelihood(Pop2 and Pop3) | 0.8228 | 0.0006 (***) |
| Mean index of genotype likelihood(Pop3 and Pop1) | 0.8637 | 0.0002 (***) |
| Mean index of genotype likelihood(Pop3 and Pop2) | 0.7043 | 0.0002 (***) |
