# Supplementary material for: The Phytogeographic History of Common Walnut in China
Source: Front Plant Sci. 2018 Sep 21;9:1399. doi: 10.3389/fpls.2018.01399 (PMC6160591; doi:10.3389/fpls.2018.01399)
Supplement: TABLE S7 — Summary of 19 bioclimatic layers used in niche models. [file Table_7.DOC]

**Table S7**. Summary of 19 bioclimatic layers used in niche models.

| varible |  | Percent contribution |
| --- | --- | --- |
| BIO1 | Annual Mean Temperature | 3.2% |
| BIO2 | Mean Diurnal Range (Mean of monthly (max temp - min temp)) | 3.4% |
| BIO3 | Isothermality (BIO2/BIO7) (* 100) | 4.2% |
| BIO4 | Temperature Seasonality (standard deviation *100) | 3.1% |
| BIO5 | Max Temperature of Warmest Month | 2.1% |
| BIO6 | Min Temperature of Coldest Month | 4.6% |
| BIO7 | Temperature Annual Range (BIO5-BIO6) | 1.6% |
| BIO8 | Mean Temperature of Wettest Quarter | 1.4% |
| **BIO9** | **Mean Temperature of Driest Quarter** | **21.6%** |
| BIO10 | Mean Temperature of Warmest Quarter | 1.3% |
| **BIO11** | **Mean Temperature of Coldest Quarter** | **10.4%** |
| BIO12 | Annual Precipitation | 2.7% |
| BIO13 | Precipitation of Wettest Month | 2.3% |
| BIO14 | Precipitation of Driest Month | 0.9% |
| **BIO15** | **Precipitation Seasonality (Coefficient of Variation)** | **15.1%** |
| BIO16 | Precipitation of Wettest Quarter | 1.0% |
| BIO17 | Precipitation of Driest Quarter | 0.7% |
| **BIO18** | **Precipitation of Warmest Quarter** | **16.9%** |
| BIO19 | Precipitation of Coldest Quarter | 3.5% |
